# A Rigid-Body Pendulum Model for Plyometric Push-Up Biomechanics: Analytical Derivation and Numerical Quantification of Flight Time, Arc Displacement, Maximum Height, and Mechanical Power Output

**DOI:** 10.3390/bioengineering13040445

**Published:** 2026-04-11

**Authors:** Wissem Dhahbi

**Affiliations:** Research Unit “Sport Sciences, Health and Movement” (UR22JS01), High Institute of Sport and Physical Education of Kef, University of Jendouba, Kef 7100, Tunisia; wissem.dhahbi@gmail.com

**Keywords:** angular velocity, athletic performance, body weight support, conservative mechanical system, energy conservation, musculoskeletal modeling, rotational kinematics, stretch-shortening cycle

## Abstract

Aim: Conventional free-fall kinematic models applied to plyometric push-up assessment treat the upper body as a vertically translating point mass, ignoring the curvilinear trajectory imposed by the ankle pivot and systematically biasing flight-time and height estimates. Methods: A planar rigid-body pendulum pivoting about the ankle axis was formulated via two independent derivation pathways (static moment equilibrium and a gravitational-torque coordinate approach), yielding effective pendulum length L = (M_W_/M) × L_OS_. Closed-form expressions for flight time, arc displacement, maximum height, and mean mechanical power were derived analytically from energy conservation and compared against free-fall predictions across seven pendulum arm lengths (L_OW_ = 0.50–2.00 m) and 500 initial hand velocities per length, using adaptive Gauss–Kronrod quadrature (relative tolerance 10^−10^) with ODE cross-validation (maximum discrepancy < 2.5 × 10^−7^ s). Results: Flight time equivalence (t^H^ = t^G^) was formally established. The free-fall model overestimated flight time by up to 18.82% (Δt = 0.096 s; L_OW_ = 0.50 m, V_H,0_ = 2.50 m/s) and maximum height by up to 28.43% (Δh = 0.087 m; L_OW_ = 0.50 m, t_flight_ = 0.50 s), with both errors growing nonlinearly with initial velocity. Overestimation in height was proportionally larger at shorter pendulum arm lengths (18.18% at t_flight_ = 0.30 s for L_OW_ = 0.50 m vs. 10.91% for L_OW_ = 1.00 m). Conclusions: The pendulum model provides a physically consistent, analytically tractable framework for geometry-adjusted upper-body power assessment from four field-obtainable anthropometric inputs. These results reflect computational self-consistency; prospective experimental validation against force-plate kinematics is required before applied deployment. Prospective empirical validation against dual force-plate and motion-capture reference data is required to establish the model’s accuracy boundaries under real push-up kinematics.

## 1. Introduction

Upper-body muscular power is a fundamental determinant of athletic performance across a wide spectrum of sports, including combat sports, swimming, throwing events, gymnastics, and rugby, and its accurate assessment carries direct implications for talent identification, training program evaluation, and fatigue monitoring in competitive populations [1,2,3,4]. In contrast to the extensive body of literature characterizing lower-body explosive capacity through vertical jump testing, the evaluation of upper-body power remains methodologically less mature [4], constrained by a comparatively smaller number of validated assessment tools and by longstanding conceptual inconsistencies in how those tools are applied and interpreted [5]. The principal challenge is the absence of a standardized, field-deployable test that is simultaneously physically valid, analytically tractable, and free of systematic measurement bias [4,6]. Addressing that challenge requires not only sound instrumentation but also a mechanically accurate kinematic model capable of translating raw observational data, such as flight time, into performance indices that correctly represent the underlying physics of upper-body ballistic motion.

The most widely established methods for assessing upper-body explosive power are the seated medicine ball throw, the bench press throw, and, more recently, the ballistic push-up (BPU) [7]. Each presents logistical or conceptual limitations. The medicine ball throw requires an arbitrary choice of implement mass and has demonstrated modest correlations with force-plate-derived power in certain populations [8,9]. The bench press throw is typically executed in a Smith machine and requires a Smith machine fixture and continuous spotter presence, substantially restricting its use in large-scale field testing [10,11]. The BPU has attracted growing attention precisely because it requires no specialized equipment beyond a force plate or contact mat, recruits the same musculature as the bench press, and generates a measurable flight phase during which both hands lose contact with the ground simultaneously [12]. Wang et al. [8] demonstrated that force-time-derived performance measures from the BPU, including peak force, mean force, net impulse, and peak velocity, exhibited moderate to very high test–retest reliability (ICC = 0.849–0.971) across 60 recreationally active men, and that BPU-derived mean force predicted one-repetition maximum (1RM) bench press with R^2^ = 0.837. Bartolomei et al. [10] subsequently confirmed that the BPU provides power estimates comparable to those of the bench press throw, with very large to extremely large correlations (r = 0.70–0.89) between the two methods, without requiring a Smith machine or spotter infrastructure.

The biomechanical complexity of the plyometric push-up fundamentally distinguishes it from vertical jump-based assessment paradigms [13]. Unlike the vertical jump, where the center of mass follows a predominantly rectilinear trajectory under gravitational deceleration, the plyometric push-up involves segmental rotation about a fixed ankle pivot, such that the hands, the shoulders, and the center of mass all trace curvilinear arcs throughout the flight phase. This constraint imposes pendular mechanics on the motion, rendering the equations of purely vertical ballistic flight physically inappropriate for quantifying hand displacement, maximum height, and take-off velocity from a measured flight time. Additionally, the stretch-shortening cycle (SSC) dynamics of the upper extremities differ from their lower-body counterparts due to distinct muscle architecture, neural activation patterns, and the contribution of trunk and core musculature to proximal force transmission, all of which introduce analytical complexities absent from conventional jump models [12,14,15].

Despite these recognized characteristics, the kinematic model universally applied to BPU flight-phase scoring treats the body as a freely falling point mass, yielding t_flight_ = 2 V_0_/g and h_max_ = V_0_^2^/2 g. This free-fall approach assumes rectilinear vertical motion and requires no geometric inputs, but introduces a structural overestimation error that grows nonlinearly with initial velocity by disregarding the curvilinear, rotationally constrained trajectory of the push-up. Dual force-plate methods provide model-independent ground reaction force data and constitute the current empirical reference standard, but require two synchronized platforms and are not field-deployable for routine assessment [16,17]. The pendulum model proposed here assumes a rigid body rotating about a fixed ankle pivot in the sagittal plane, requires four anthropometric inputs, and corrects the geometric source of the free-fall error analytically [8]; its principal current limitation is the absence of prospective experimental validation against empirical kinematic data [14].

Despite these recognized limitations, no published study has developed a formally derived mechanical model that replaces the free-fall simplification with a physically appropriate description of the rotational flight-phase trajectory of the plyometric push-up. The present study addresses this gap by formulating a rigid-body pendulum model that treats the body as a single-link pendulum pivoting about the ankle. Specifically, this investigation aimed to: (i) derive the effective pendulum length from two independent anthropometric frameworks and establish their mathematical equivalence; (ii) determine the flight time of the hands and the whole-body center of mass through analytically derived and numerically verified expressions; (iii) calculate the arc displacement of the hands and center of mass along their respective circular trajectories; (iv) quantify the maximum vertical height reached by both reference points from the measured take-off velocity; and (v) establish and numerically quantify the systematic divergence between free-fall and pendulum model predictions of flight time and maximum height across the full physiologically admissible parameter space.

## 2. The Pendulum Model: Conceptualization and Geometric Framework

### 2.1. Model Conceptualization and Biomechanical Rationale

The mechanical analysis of a plyometric push-up presents a fundamental challenge that differentiates it from lower-body plyometric assessments. In vertical jump protocols, the body’s center of mass (CoM) undergoes ballistic displacement oriented along the gravitational axis, allowing flight-phase kinematics to be modeled by equations of uniformly accelerated rectilinear motion [18]. In a plyometric push-up, the upper body does not translate vertically as a free-falling point mass; rather, the body rotates about the ankle joint complex (point O), and the CoM traverses a curvilinear, arc-like path in the sagittal plane. The application of conventional free-fall kinematic models introduces systematic overestimations of flight time and maximum height, because those models disregard the continuous exchange between kinetic and potential energy that characterizes rotational dynamics.

**Figure 1:**
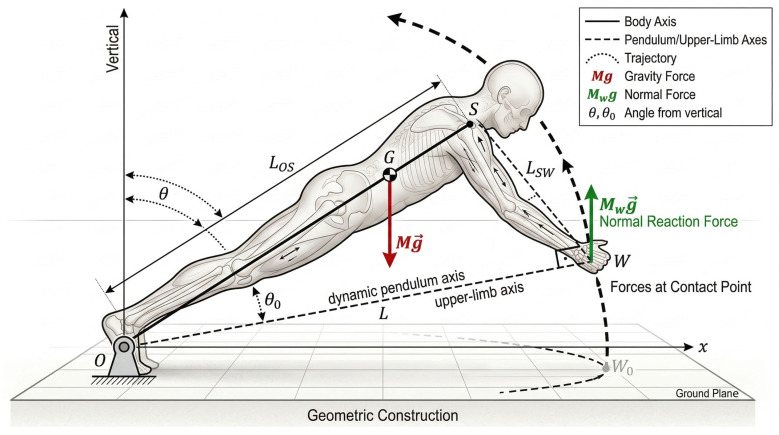
Planar rigid-body pendulum model of the plyometric push-up: sagittal-plane geometric framework defining system parameters, segment lengths, and initial kinematic configuration. Schematic representation of the planar inverted-pendulum model in the initial static push-up configuration. The ankle joint complex serves as the fixed pivot point O; the acromion process of the shoulder defines point *S*; and the center of the wrist joint defines the hand contact point W. The global center of mass *G* lies along the pendulum axis O–S at the effective pendulum length $L=\left(M_{W}/M\right)\;L_{OS}$ from O (Equation (6)). The segment length $L_{OS}$ (m) denotes the straight-line distance from O to S along the body’s longitudinal axis; $L_{SW}$ (m) denotes the straight-line distance from S to W with the arm extended perpendicularly to the supporting surface. The hand-to-pivot distance $L_{OW}=\sqrt{L_{OS}^{2}-L_{SW}^{2}}$ (m) constitutes the radius of the circular arc traversed by W during the flight phase (Equation (18)). The initial pendulum angle $\theta_{0}=arcsin\left(L_{SW}/L_{OS}\right)$ (rad) defines the inclination of the pendulum axis above the horizontal in the starting configuration (Equation (17)). The coordinate origin is located at O, with the positive x-axis directed horizontally toward W and the positive y-axis directed vertically upward. All labeled quantities are primary inputs to the performance index derivations of Section 3.

The present framework conceptualizes the human body during a plyometric push-up as a rigid planar pendulum pivoting about the fixed ankle axis, point O. The effective pendulum arm connects O to the global CoM (point G) with length L (m). The hand contact point W travels along a distinct arc of radius L_OW_ (m). The flight phase is initiated at the instant the vertical ground reaction force under the hands falls to zero, at which moment the system possesses an angular velocity ω_O_ (rad/s) about O. The subsequent motion is treated as that of a conservative rigid pendulum, with gravitational potential energy governing the trajectory until the hands return to the ground (Figure 1).

### 2.2. Anthropometric Measurements and Body Segmentation

Parameterization of the pendulum model requires four primary measurements. Total body mass M (kg) is recorded with the subject standing in the anatomical position. In the static push-up position (arms fully extended and perpendicular to the supporting surface), three additional quantities are obtained: (i) shoulder height L_OS_ (m)—the straight-line distance from the ankle axis O to the acromion process S; (ii) upper-limb length L_SW_ (m)—the distance from the acromion process S to the center of the wrist joint W; and (iii) hand-supported mass M_W_ (kg)—the mass measured by a calibrated scale positioned beneath the hands in static equilibrium. These four quantities collectively determine all governing geometric and inertial parameters of the model.

### 2.3. Determination of the Effective Pendulum Length (L)

Two independent derivations of the effective pendulum length L are presented below, followed by a formal reconciliation of their results.

#### 2.3.1. Static Equilibrium Approach

In the initial static push-up position, the system is in complete rotational equilibrium about O. Three external forces act: the ground reaction force at O (generating no moment about O), the total body weight Mg acting vertically downward through G, and the vertical reaction force M_W_g acting vertically upward at W. The vector moment equation about O in the sagittal plane is:

(1) $$\sum {\vec{M}}_{O}=\vec{OG}\times \left(-Mg\;\hat{j}\right)+\vec{OW}\times \left(M_{W}g\;\hat{j}\right)=\vec{0}$$

With the coordinate origin at O, the position vectors are:

(2) $$\vec{OG}=\left(\begin{array}{c} Lcos\theta_{0}\\ Lsin\theta_{0}\\ 0 \end{array}\right),\;\;\vec{OW}=\left(\begin{array}{c} L_{OS}cos\theta_{0}\\ 0\\ 0 \end{array}\right)$$

Computing the z-component of each cross product:

(3) $${\left(\vec{OG}\times \left(-Mg\;\hat{j}\right)\right)}_{z}=-MgLcos\theta_{0}$$

(4) $${\left(\vec{OW}\times \left(M_{W}g\;\hat{j}\right)\right)}_{z}=M_{W}g\;L_{OS}cos\theta_{0}$$

Setting the sum of *z*-moments to zero:

(5) $$-MgLcos\theta_{0}+M_{W}g\;L_{OS}cos\theta_{0}=0$$

Since cos θ_0_ > 0 for any physiologically admissible push-up configuration (i.e., θ_0_ < π/2), this factor cancels throughout, yielding:

(6) $$L=\frac{M_{W}}{M}\;L_{OS}$$

#### 2.3.2. Center-of-Mass Coordinate Approach

As an independent derivation, the position of G is estimated via a two-point mass model. The total body mass is distributed between the ankle pivot O, bearing the residual mass M_f_ = M − M_W_ (kg), and the hand contact point W, bearing the measured mass M_W_. In the world coordinate frame (origin at O, x-axis horizontal, y-axis vertical), both mass points lie at ground level—O at (0, 0) and W at (L_OW_, 0), where L_OW_ denotes the horizontal distance from O to W (established as L_OW_ = L_OS_ cos θ_0_ in Section 2.4). The Cartesian coordinates of the resulting CoM are:

(7) $$x_{G}=\frac{M_{W}\;L_{OW}}{M}=\frac{M_{W}\;L_{OS}cos\theta_{0}}{M}$$

(8) $$y_{G}=\frac{M_{f}\cdot 0\;{+M}_{W}\cdot 0}{M}=0$$

The Euclidean distance from O to the computed CoM is therefore:

(9) $$d_{OG}=\sqrt{x_{G}^{2}+y_{G}^{2}}=\frac{M_{W}\;L_{OS}cos\theta_{0}}{M}$$

To recover the effective pendulum length from this two-point model, the governing criterion must be gravitational torque equivalence (i.e., the condition that the torque produced about O by the two-point mass system equals that of the actual distributed body) rather than geometric distance. The total gravitational torque about O (in the z-direction) produced by the two-point mass system is:

(10) $$\tau_{grav}=M_{W}\;g\;L_{OS}cos\theta_{0}$$

For a simple pendulum of total mass M and effective length L_eff_ inclined at angle θ_0_ from horizontal, the gravitational torque about O is M g L_eff_ cos θ_0_. Setting this equal to expression (10) and canceling g cos θ_0_:

(11) $$L_{eff}=\frac{M_{W}}{M}\;L_{OS}$$

Equations (6) and (11) are identical, confirming the self-consistency of both approaches.

#### 2.3.3. Equivalence and Reconciliation

Both derivation pathways operationalize the same static rotational equilibrium condition about O, so their convergence is mathematically consistent rather than independently confirmatory. Both yield:

(12) $$L=L_{eff}=\frac{M_{W}}{M}\;L_{OS}$$

In practical terms, L scales linearly with the hand-supported mass fraction M_W_/M and the shoulder height L_OS_; for a subject supporting 55% of body mass at the hands with L_OS_ = 1.10 m, L ≈ 0.60 m, which governs all subsequent energy-conservation derivations. A precise conceptual distinction must, however, be drawn between this effective length and the Euclidean distance d_OG_ obtained from Equation (9). Comparing Equations (9) and (12):

(13) $$d_{OG}=\frac{M_{W}\;L_{OS}cos\theta_{0}}{M}=Lcos\theta_{0}$$

The Euclidean distance d_OG_ is smaller than L by the factor cos θ_0_. The origin of this discrepancy lies in the geometric constraint imposed by the two-point mass model: by placing both mass points at y = 0 (ground level), the model necessarily produces y_G_ = 0, forcing the computed CoM onto the horizontal axis. The true CoM G, however, lies along the inclined pendulum axis O-S at height L sin θ_0_ = (M_W_/M) L_OS_ sin θ_0_ > 0. The two-point model therefore correctly reproduces the horizontal projection of G (Equation (7) recovers x_G_ = L cos θ_0_ = d_OG_), but systematically underestimates the height of G.

The physical resolution is that L is not the Euclidean distance from O to G; it is the torque-equivalent pendulum length, defined as the ratio of gravitational torque to total gravitational force:

(14) $$L\equiv \frac{\tau_{grav}}{M\;g\;cos\theta_{0}}=\frac{M_{W}\;g\;L_{OS}cos\theta_{0}}{M\;g\;cos\theta_{0}}=\frac{M_{W}\;L_{OS}}{M}$$

This definition is the mechanically operative one: it is the quantity L that appears in the equation of angular motion $I\ddot{\theta }=-MgLcos\theta$ and in all subsequent energy conservation derivations. The two-point mass model is therefore a valid mechanical surrogate for torque analysis, not a geometric surrogate for locating G in three-dimensional space. In practice, the deviation between d_OG_ and L is proportional to (1 – cos θ_0_), which is less than 4\% for θ_0_ ≤ 16° (since cos 16° ≈ 0.961), the range encompassing the vast majority of adult push-up configurations. Throughout all subsequent derivations, L = (M_W_/M) L_OS_ is adopted as the governing dynamic parameter.

It must be noted that L is the torque-equivalent simple pendulum length, not the equivalent length for a compound pendulum of distributed mass. The dynamically correct equivalent pendulum length is: $L_{\mathrm{eq}}\;=\;\frac{I_{O}}{M\cdot L_{\mathrm{CoM}}}$ and $I_{O}\;=\;I_{\mathrm{CoM}}\;+\;M\cdot {L_{\mathrm{CoM}}}^{2}$.

### 2.4. Determination of the Initial Pendulum Angle (θ_0_)

The initial angle θ_0_ characterizes the orientation of the pendulum axis (line O-G, collinear with line O-S) relative to the horizontal plane in the starting push-up configuration. In the static push-up position, the arms are extended perpendicularly to the supporting surface, so that the wrist W lies directly below the shoulder S. The shoulder S is located at world coordinates (L_OS_ cos θ_0_, L_OS_ sin θ_0_). Because the arms are vertical, the wrist W is displaced vertically below S by the distance L_SW_, giving:

(15) $$W=\left(L_{OS}cos\theta_{0},\;L_{OS}sin\theta_{0}-L_{SW}\right)$$

For W to lie at ground level (i.e., y_W_ = 0, as required by the physical setup), the vertical coordinate must vanish:

(16) $$L_{OS}sin\theta_{0}-L_{SW}=0$$

Solving for θ_0_:

(17) $$\theta_{0}=arcsin\left(\frac{L_{SW}}{L_{OS}}\right)$$

where the argument is defined for L_SW_ < L_OS_, which is always satisfied for physiologically normal proportions. This relation establishes the geometric consistency of the model: the perpendicularity of the arms to the floor, combined with the anthropometric lengths L_OS_ and L_SW_, uniquely determines the initial orientation of the pendulum. From Equation (16), the horizontal distance from O to W follows directly:

(18) $$L_{OW}=L_{OS}cos\theta_{0}=L_{OS}\sqrt{1-{\left(\frac{L_{SW}}{L_{OS}}\right)}^{2}}=\sqrt{L_{OS}^{2}-L_{SW}^{2}}$$

The quantity L_OW_ is the radius of the arc traversed by the hands during the flight phase and appears in all hand-referenced performance indices derived in Section 3.2.

### 2.5. Model Assumptions and Simplifications

The analytical tractability of the pendulum model is contingent upon the following idealizing conditions, which collectively define its domain of validity.

*Rigid Body:* The human body is modeled as a single rigid segment throughout the flight phase. During push-off, inter-segmental rotation at the elbow, shoulder, and hip contributes to force generation; the rigid-body constraint applies exclusively from the instant of hand take-off to hand landing, the interval governed by the conservative equations of Section 3. This assumption is most closely approached in trained individuals who maintain strict whole-body tension throughout the flight phase [19]. In recreational athletes, pronounced hip flexion or segmental motion during flight produces deviations from the predicted pendular arc that the present model cannot quantify. As a first-order illustration, a 5° shoulder-flexion perturbation during flight displaces the CoM from the nominal pendular arc by approximately L·cos (θ_0_)·δ ≈ 0.05 m for representative anthropometry (L = 0.60 m, θ_0_ = 12°), a displacement of comparable order to the height discrepancy between models at short flight times (Δh = 0.020 m; t_flight_ = 0.30 s, L_OW_ = 0.50 m). Precise quantification of inter-segmental deviation effects on t_H_ and h_max_ requires prospective motion-capture measurements and is registered as a priority for future work; for non-rigid subjects, model outputs should be interpreted as upper bounds on the pendulum-consistent performance estimate.

*Planar Motion:* All motion is restricted to the sagittal plane. Lateral displacement, axial rotation, and inter-limb asymmetry are excluded. The assumption is justified by the bilateral symmetry of standard push-up mechanics and is supported by the planar trajectory of the CoM observed in controlled laboratory conditions [19].

*Fixed Pivot at the Ankles:* The ankle joint complex (O) is treated as a frictionless, fixed pivot throughout the motion. No translational displacement of O is permitted in either phase. The ground reaction force at O passes through the pivot and generates no moment about it. Minor translational displacement of the ankle at takeoff, which is more common in recreational than in trained push-up execution, would alter the effective pivot location and introduce a position-dependent error in L that the present model does not accommodate.

*Arms Perpendicular to the Supporting Surface:* The initial configuration requires the arms to be fully extended and oriented vertically (perpendicular to the floor). This constraint is necessary to establish the geometric relation sin θ_0_ = L_SW_/L_OS_ (Equation (17)) and to ensure that W lies at ground level at the moment of hand take-off.

*Two-Point Mass Distribution:* For the purposes of locating the effective CoM, total body mass is distributed between O (mass M_f_ = M − M_W_) and W (mass M_W_). This simplification is mechanically valid for gravitational torque analysis (Section 2.3). It does not account for the distributed inertial properties of the body (Section 2.3.3); the negligible-error claim holds only for moment-arm calculations, not for oscillation-period predictions where IO governs.

*Conservative Flight Phase:* Aerodynamic drag, limb-damping torques, and any dissipative effects are considered negligible during the flight phase. Aerodynamic drag, limb-damping torques, and any dissipative effects are considered negligible during the flight phase [20]. For a body mass of 75 kg at V_H_, 0 ≤ 3.0 m/s, the peak aerodynamic drag force $F_{d}\;\approx \;\frac{1}{2}\rho \cdot C_{d}\cdot A\cdot v^{2}\;\approx \;0.5\;N$. The system is treated as mechanically conservative from the moment of hand take-off to the moment of hand landing, permitting the direct application of energy conservation throughout Section 3.

*Small Difference Between L_OW_ and L: * For the purpose of the quarter-period approximation used in power derivations (Section 3.2.4 and Section 3.3.4), the push-off duration is estimated from the linear small-amplitude pendulum period. This approximation introduces errors that grow with initial amplitude; the precise push-off duration for large initial velocities requires numerical integration, as addressed in Section 4.

## 3. Biomechanical Derivations

### 3.1. Kinematic Equivalence: Flight Time of the Hands (t_H_) and Center of Mass (t_G_)

Because the body is modeled as a rigid pendulum rotating about fixed pivot O, every point shares the same instantaneous angular velocity ω (rad/s). The linear velocity of any point P at Euclidean distance r from O is:

(19) $$V_{P}=\omega \cdot r$$

directed tangentially to the arc of radius r. Applying this to the hands (W, at distance L_OW_ from O) and to the CoM (G, at distance L from O) at the instant of hand take-off:

(20) $$V_{H,0}=\omega_{0}\cdot L_{OW}$$

(21) $$V_{G,0}=\omega_{0}\cdot L$$

Dividing Equation (21) by Equation (20):

(22) $$V_{G,0}=V_{H,0}\cdot \frac{L}{L_{OW}}$$

Substituting L = (M_W_/M) L_OS_ (Equation (12)) and L_OW_ = L_OS_ cos θ_0_ (Equation (18)):

(23) $$V_{G,0}=V_{H,0}\cdot \frac{M_{W}}{Mcos\theta_{0}}$$

Equation (23) relates the directly measurable initial hand velocity V_H,0_ to the initial CoM velocity V_G,0_, which governs the global energetics of the system. Because the ratio M_W_/(M cos θ_0_) is always less than unity for physiologically normal mass distributions (M_W_ < M) and push-up angles (cos θ_0_ < 1 but M_W_/M < cos θ_0_ typically), V_G,0_ is in practice smaller than V_H,0_.

Since the system is rigid and rotates about a fixed pivot, the angular equation of motion governing the flight phase is identical regardless of which point on the pendulum is used as a reference. The angular displacement traversed from take-off (angle θ_0_ for G, angle φ_0_ = 0 for W) to the corresponding maximum and back to the initial angle is the same for all body points in terms of elapsed time. Consequently, the time interval between hand take-off and hand landing, designated t_H_ (s), is equal to the corresponding flight time of the CoM, t_G_ (s):

(24) $$t_{H}=t_{G}$$

This identity is of direct practical importance: t_H_ is measurable with standard force-plate instrumentation (as the duration of the off-plate interval) or with contact mats, and Equation (24) confirms that this measurement can be used without modification in the CoM-referenced performance equations of Section 3.3. No separate kinematic tracking of the CoM is required.

### 3.2. Performance Indices for the Hands (Option 1)

The following performance indices characterize the flight phase from the perspective of the hands (W), treated as the end-effector of the pendulum of arm length L_OW_. The angle φ (rad) denotes the angle of the arm O-W above the horizontal, measured positive upward. In the initial position, φ = 0 (W at ground level) and the tangential velocity of W is V_H,0_. At maximum displacement, φ = φ_max,H_ and the instantaneous velocity of W is zero. The height of W above its initial position at angle φ is h_W_(φ) = L_OW_ sin φ.

#### 3.2.1. Maximum Angular Displacement of the Hands (φ_max,H_)

Applying conservation of mechanical energy between the take-off state (φ = 0, V = V_H,0_) and the state of maximum displacement (φ = φ_max,H_, V = 0), and noting that the height gain of W is L_OW_ sin φ_max,H_:

(25) $$1/2M_{W}V_{H,0}^{2}=M_{W}\;g\;L_{OW}sin\varphi_{max,H}$$

Solving:

(26) $$\varphi_{max,H}=arcsin\left(\frac{V_{H,0}^{2}}{2\;g\;L_{OW}}\right)$$

For Equation (26) to yield a physically admissible solution, the condition $V_{H,0}^{2}\leq 2gL_{OW}$ must be satisfied, which corresponds to the physical requirement that W does not reach or exceed the height of the pivot O during flight. For representative anthropometric values (L_OW_ ≈ 0.7–0.9 m), this upper bound corresponds to V_H,0_ ≤ 3.7–4.2 m/s, which encompasses all reported push-up take-off velocities in the literature. An equivalent expression in terms of the measurable system parameters follows directly by substituting $L_{OW}=\sqrt{L_{OS}^{2}-L_{SW}^{2}}$:

(27) $$\varphi_{max,H}=arcsin\left(\frac{V_{H,0}^{2}}{2\;g\;\sqrt{L_{OS}^{2}-L_{SW}^{2}}}\right)$$

#### 3.2.2. Arc Displacement of the Hands (S_hand_)

During the ascending half of the flight phase, the hands traverse a circular arc of radius LOW centered at O, from φ = 0 to φ = φ_max,H_. The total arc length (expressed with φ_max_, H in radians) is:

(28) $$S_{hand}=L_{OW}\cdot \varphi_{max,H}$$

Substituting Equation (26):

(29) $$S_{hand}=L_{OW}\cdot arcsin\left(\frac{V_{H,0}^{2}}{2\;g\;L_{OW}}\right)$$

This parameter quantifies the total curvilinear path of the hands along the pendular arc. It is distinct from the maximum vertical height h_max,H_ (Section 3.2.3) and constitutes a spatial performance descriptor that is inaccessible to free-fall models, which implicitly assume rectilinear vertical displacement. 

#### 3.2.3. Maximum Vertical Height of the Hands (h_max,H_)

The maximum vertical displacement of W above its starting position (ground level) is obtained directly from the height function h_W_ = L_OW_ sin φ:

(30) $$h_{max,H}=L_{OW}sin\varphi_{max,H}$$

Substituting sin φ_max,H_ from Equation (25):

(31) $$h_{max,H}=\frac{V_{H,0}^{2}}{2\;g}$$

Equation (31) is formally identical in structure to the free-fall kinematic relation h = V_0_^2^/(2 g). This formal equivalence does not, however, imply that the pendulum and free-fall models yield the same predictions when flight time t_H_ is used as the experimental input, because the relationship between V_H,0_ and t_H_ differs fundamentally between the two models. In the free-fall model, V_0_ = gt_H_/2 follows directly from constant-acceleration kinematics, giving h_max,FF_ = gt_H_^2^/8. In the pendulum model, the relationship between V_H,0_ and t_H_ is governed by the nonlinear pendulum equation of motion and requires numerical integration (Section 4.1). The substitution of the free-fall velocity-time relationship into Equation (31) would reproduce the free-fall height formula and therefore offer no improvement over the simpler model. The proper usage of Equation (31) is in conjunction with the pendulum-derived value of V_H,0_, as established numerically in Section 4.

#### 3.2.4. Mechanical Power Output at the Hands (P_hand_)

The following derivation constitutes a first-order analytical estimate, valid in the small-angle limit (φ_max,H_ ≪ 1 rad); it is provided for reference and comparative purposes, not as a robust field-applicable output under large-amplitude conditions. The mean mechanical power developed by the musculoskeletal system during the push-off phase, referenced to the hands, is defined as the ratio of net mechanical work performed to the duration of the push-off phase. The push-off phase begins with the system at rest in the initial push-up position and concludes at the instant of hand take-off, when the hands possess kinetic energy ½ M_W_·V_H,0_. The total mechanical work performed by the muscles is therefore:

(32) $$W_{hand}=1/2M_{W}\;V_{H,0}^{2}$$

This equals the mechanical energy stored in the hand-mass subsystem at take-off, consistent with the work-energy theorem applied from the rest state to the take-off instant.

The duration of the push-off phase is equal, by time-reversal symmetry of the conservative pendulum, to the time elapsed from hand take-off to the moment of maximum hand height, denoted t_push,H_. For a pendulum of arm length L_OW_ executing small-amplitude oscillations, this duration is approximated by the quarter-period of the linearized (simple harmonic) system:

(33) $$t_{push,H}\approx \frac{\pi }{2}\sqrt{\frac{L_{OW}}{g}}$$

The mean mechanical power output at the hands is:

(34) $$P_{hand}=\frac{W_{hand}}{t_{push,H}}=\frac{M_{W}\;V_{H,0}^{2}}{\pi }\sqrt{\frac{g}{L_{OW}}}$$

It is noted that Equation (33) constitutes a small-amplitude approximation, valid strictly for φ_max,H_ ≪ 1 rad. For subjects with high take-off velocities, where φ_max,H_ is not negligible, the exact push-off duration departs from this estimate and must be evaluated by numerical integration of the nonlinear pendulum equation (Section 4.1). The use of Equation (34) under large-amplitude conditions will overestimate mean power output to the extent that the true push-off duration exceeds $(\pi /2)\sqrt{L_{OW}/g}$.

### 3.3. Performance Indices for the Center of Mass (Option 2)

The following performance indices characterize the flight phase from the perspective of the CoM (G), treated as the mass point of the pendulum of arm length L. The angle θ (rad) denotes the angle of the pendulum arm O-G above the horizontal, measured positive upward. In the initial position, θ = θ_0_ > 0 and the tangential velocity of G is V_G,0_. At maximum displacement, θ = θ_max_ and the instantaneous velocity of G is zero. The height of G above its initial position at angle θ is h_G_(θ) = L (sin θ – sin θ_0_).

#### 3.3.1. Maximum Angular Displacement of the CoM (θ_max_)

Applying conservation of mechanical energy between the take-off state (θ = θ_0_, V = V_G,0_) and the state of maximum displacement (θ = θ_max_, V = 0):

(35) $$1/2M\;V_{G,0}^{2}=M\;g\;L\left(sin\theta_{max}-sin\theta_{0}\right)$$

Solving for θ_max_:

(36) $$\theta_{max}=arcsin\left(\frac{V_{G,0}^{2}}{2\;g\;L}+sin\theta_{0}\right)$$

Substituting V_G,0_ = V_H,0_·L/L_OW_ from Equation (22) and L/L_OW_ = M_W_/(M cos θ_0_) from Equations (12) and (18):

(37) $$\theta_{max}=arcsin\left(\frac{V_{H,0}^{2}\;M_{W}^{2}}{2\;g\;M^{2}\;L_{OS}{cos}^{2}\theta_{0}}+sin\theta_{0}\right)$$

For Equation (36) to be physically admissible, the constraint V_G,0_^2^/(2 gL) +sinθ_0_ ≤ 1 must hold, which sets an upper bound on initial angular velocity corresponding to the physical limit at which G reaches the vertical through O. This constraint is expressed as:

(38) $$V_{G,0}\leq \sqrt{2\;g\;L\left(1-sin\theta_{0}\right)}$$

The initial elevation sin θ_0_ in Equation (36) elevates the maximum reachable angle relative to the case θ_0_ = 0; for a given V_G,0_, the CoM attains a higher angular position when it begins at a positive initial angle, because part of the potential energy budget is already stored in the initial configuration.

#### 3.3.2. Arc Displacement of the Center of Mass (S_G_)

During the ascending half of the flight phase, the CoM traverses a circular arc of radius L centered at O, from angle θ_0_ to θ_max_. The total arc length (with angles in radians) is:

(39) $$S_{G}=L\left(\theta_{max}-\theta_{0}\right)$$

Substituting from Equations (12) and (36):

(40) $$S_{G}=\frac{M_{W}\;L_{OS}}{M}\left[arcsin\left(\frac{V_{G,0}^{2}}{2\;g\;L}+sin\theta_{0}\right)-\theta_{0}\right]$$

This parameter quantifies the total curvilinear displacement of the CoM along its pendular arc, integrating both angular amplitude and pendulum geometry into a single spatial metric. Because S_G_ < h_max_ is not guaranteed (the arc length along a large-radius, small-angle pendulum can exceed the corresponding vertical rise), S_G_ and h_max_ provide complementary rather than redundant spatial descriptors.

#### 3.3.3. Maximum Vertical Height of the CoM (h_max,G_)

The maximum vertical rise of G above its take-off position is:

(41) $$h_{max,G}=L\left(sin\theta_{max}-sin\theta_{0}\right)$$

Substituting sin θ_max_ from Equation (35):

(42) $$h_{max,G}=L\left[\frac{V_{G,0}^{2}}{2\;g\;L}+sin\theta_{0}-sin\theta_{0}\right]=\frac{V_{G,0}^{2}}{2\;g}$$

Expressed entirely in terms of the directly measurable hand take-off velocity V_H,0_ and system parameters, by substituting V_G,0_ = V_H,0_ L/L_OW_:

(43) $$h_{max,G}=\frac{V_{H,0}^{2}\;L^{2}}{2\;g\;L_{OW}^{2}}=\frac{M_{W}^{2}\;V_{H,0}^{2}}{2\;g\;M^{2}{cos}^{2}\theta_{0}}$$

Equation (43) differs from the hand-referenced height h_max,H_ (Equation (31)) by the factor (L/L_OW_)^2^ = (M_W_/(M cos θ_0_))^2^. For representative adult values of M_W_/M ≈ 0.50–0.60 and θ_0_ ≈ 10° − 15° (cos θ_0_ ≈ 0.966 − 0.985), this factor ranges from approximately 0.26 to 0.39, confirming that the CoM rises to a substantially smaller height than the hands during the flight phase, consistent with the mechanical expectation that distal points of a pendulum execute larger excursions than proximal ones.

#### 3.3.4. Mechanical Power Output at the Center of Mass (P_G_)

As with Section 3.2.4, the following expression is a first-order approximation applicable in the small-angle limit only; the same error bounds described in Section 3.2.4 apply. The mean mechanical power at the CoM level provides a global performance metric integrating the full system mass and CoM kinematics. The net mechanical work performed by the musculoskeletal system on the CoM during push-off, from rest to the take-off state, is:

(44) $$W_{G}=1/2M\;V_{G,0}^{2}$$

The push-off duration at the CoM level is estimated by the quarter-period of the equivalent linearized pendulum of arm length L:

(45) $$t_{push,G}\approx \frac{\pi }{2}\sqrt{\frac{L}{g}}$$

As with Equation (33), the quarter-period approximation in Equation (45) applies to small angular amplitudes only; the same error bounds described in Section 3.2.4 apply. The mean mechanical power at the CoM is:

(46) $$P_{G}=\frac{W_{G}}{t_{push,G}}=\frac{M\;V_{G,0}^{2}}{\pi }\sqrt{\frac{g}{L}}$$

Substituting V_G,0_ = V_H,0_·L/L_OW_ and L = M_W_ L_OS_/M:

(47) $$P_{G}=\frac{M\;V_{H,0}^{2}}{\pi \;L_{OW}^{2}}\sqrt{g\;L}$$

Expanding with L = M_W_ L_OS_/M and L_OW_ = L_OS_ cos θ_0_:

(48) $$P_{G}=\frac{M_{W}\;V_{H,0}^{2}}{\pi \;L_{OS}{cos}^{2}\theta_{0}}\sqrt{\frac{g\;M_{W}\;L_{OS}}{M}}$$

The ratio PG/P_hand_ can be formed from Equations (46) and (34):

(49) $$\frac{P_{G}}{P_{hand}}=\frac{M}{M_{W}}\cdot \frac{L^{3/2}}{L_{OW}^{3/2}}$$

Since L/L_OW_ = M_W_/(Mcos θ_0_), Equation (49) reduces to:

(50) $$\frac{P_{G}}{P_{hand}}=\frac{M_{W}^{1/2}}{M^{1/2}{cos}^{3/2}\theta_{0}}$$

For typical parameter ranges, this ratio is less than unity (P_G_ < P_hand_), reflecting that the CoM-referenced power estimate accounts for a smaller effective mass moving at a smaller velocity than the hand-referenced estimate. The two power indices thus provide complementary, non-redundant perspectives on system energy output. As with Equation (34), the quarter-period approximation in Equation (45) is valid for small angular amplitudes; large initial velocities require numerical treatment of the nonlinear pendulum equation (Section 4).

## 4. Numerical Simulations

### 4.1. Flight Time Analysis: Free-Fall Model vs. Pendulum Model

#### 4.1.1. Mathematical Formulations

Two analytical models are implemented numerically to characterize and compare the flight-phase dynamics of a plyometric push-up. Both are evaluated over a systematically varied parameter space in order to quantify the divergence in their respective predictions.

*Model A: Free-Fall Model*. The hands are treated as a material point undergoing purely vertical ballistic motion under constant gravitational deceleration g. Given initial hand velocity V_H,0_ (m/s) at take-off, the flight time and maximum height are given by the closed-form kinematic expressions:

(51) $$t_{FF}=\frac{2\;V_{H,0}}{g}$$

(52) $$h_{max,FF}=\frac{V_{H,0}^{2}}{2\;g}$$

*Model B: Pendulum Model.* The hands constitute the end-effector of a rigid pendulum of arm length L_OW_ (m) pivoting about the fixed ankle axis O. The angle φ (rad) denotes the inclination of arm O-W above the horizontal. The angular equation of motion during the conservative flight phase is:

(53) $$\ddot{\varphi }+\frac{g}{L_{OW}}cos\varphi =0$$

From conservation of mechanical energy, the angular velocity at displacement φ satisfies:

(54) $$\varphi \left(0\right)=0,\;\;\dot{\varphi }\left(0\right)=\omega_{0}=\frac{V_{H,0}}{L_{OW}}$$

The maximum angular displacement φ_max,H_ is reached when $\dot{\varphi }=0$:

(55) $$\varphi_{max,H}=arcsin\left(\frac{V_{H,0}^{2}}{2\;g\;L_{OW}}\right)$$

The total flight time is twice the time required to ascend from φ = 0 to φ_max,H_:

(56) $$t_{H}=2\int_{0}^{\varphi_{max,H}}\frac{d\varphi }{\sqrt{\omega_{0}^{2}-\frac{2g}{L_{OW}}sin\varphi }}$$

The integrand of Equation (56) exhibits a square-root singularity at the upper limit φ → φ_max,H_, where $\dot{\varphi }\to 0$. Direct numerical quadrature applied to Equation (56) in its raw form yields unacceptable accuracy degradation near the turning point. A regularizing substitution is therefore applied. Setting:

(57) $$sin\varphi =sin\varphi_{max,H}\cdot {sin}^{2}u,\;\;u\in \left[0,\;\frac{\pi }{2}\right]$$

and differentiating:

(58) $$cos\varphi \;d\varphi =2\;sin\varphi_{max,H}sinucosu\;du$$

Using $\cos\varphi =\sqrt{1-\sin^{2}\varphi_{max}\sin^{4}u}$, the transformed integral becomes:

(59) $$t_{H}=\frac{4}{\omega_{0}}\sqrt{\frac{L_{OW}}{g}}\int_{0}^{\pi /2}\frac{sin\varphi_{max,H}sinucosu}{cos\varphi \;\sqrt{sin\varphi_{max,H}\left(1-{sin}^{2}u\right)}}\;du$$

After simplification:

(60) $$t_{H}=\frac{4}{\omega_{0}}\int_{0}^{\pi /2}\frac{\sqrt{sin\varphi_{max,H}}\;sinucosu}{cos\varphi \;\sqrt{1-{sin}^{2}u}}\;du$$

The transformed integrand is bounded and smooth on the closed interval [0, π/2], admitting accurate evaluation by standard adaptive Gauss–Kronrod quadrature (15-point rule) without regularization artifacts. As an independent cross-validation, Equation (53) is additionally solved as a first-order initial-value problem:

(61) $$\frac{d}{dt}\left(\begin{array}{c} \varphi \\ \dot{\varphi } \end{array}\right)=\left(\begin{array}{c} \dot{\varphi }\\ -\frac{g}{L_{OW}}cos\varphi \end{array}\right),\;\;\left(\begin{array}{c} \varphi \left(0\right)\\ \dot{\varphi }\left(0\right) \end{array}\right)=\left(\begin{array}{c} 0\\ \omega_{0} \end{array}\right)$$

using an adaptive step-size stiff/non-stiff solver with relative tolerance 10^−10^, with integration terminated by a zero-crossing event at φ(t) = 0, $\dot{\varphi }(t)<0$ (return of hands to ground level).

The model discrepancy in flight time is defined as:

(62) $$\Delta t=t_{FF}-t_{H}$$

Since t_FF_ consistently exceeds t_H_ for all V_H,0_ > 0 and all L_OW_ > 0 (proved by the strict convexity of the pendular arc relative to the vertical), Δt is positive definite and constitutes the absolute overestimation error incurred by the free-fall simplification.

#### 4.1.2. Numerical Implementation

The simulation was implemented in the R computing environment (version ≥ 4.3.0) using the packages listed in Table 1. Seven pendulum arm lengths were analyzed, spanning the anthropometrically relevant range:

(63) $$L_{OW}\in \{0.50,\;0.75,\;1.00,\;1.25,\;1.50,\;1.75,\;2.00\}\;m$$

This range spans the physiologically plausible interval for adult push-up geometry: the lower bound (0.50 m) corresponds to individuals with short effective arm reach or high L_SW_/L_OS_ ratios, and the upper bound (2.00 m) approximates the maximum anatomically feasible horizontal hand-to-ankle distance in a full push-up position for tall adults. The velocity grid upper bound of V_H,0_ ≤ 3.0 m/s is consistent with reported take-off velocities in trained populations [16,21]. For each value of L_OW_, the physical upper bound on the initial hand velocity is determined by the condition φ_max,H_ = π/2 (hands reaching the level of the pivot O), which from Equation (55) gives:

(64) $$V_{H,0}^{max}=\sqrt{2\;g\;L_{OW}}$$

Within the interval (0, V_H,0_^max^), a grid of N = 500 uniformly spaced velocity values was constructed, with the lower bound set to ϵ = 10^−4^ m/s to exclude the degenerate zero-velocity case. Although the mathematical upper bound $V_{H,0}^{max}=\sqrt{2\;g\;L_{OW}}$ was retained to characterize the full model domain, reported take-off velocities for plyometric push-up performance fall within V_H,0_ ≤ 3.0 m/s [8,16] and all practical performance indices should be interpreted within this physiologically admissible range. For each (L_OW_, V_H,0_) combination, the following quantities were computed: (i) t_FF_ via Equation (51). (ii) t_H_ via adaptive Gauss–Kronrod quadrature of the singularity-regularized integral (Equations (59) and (60)), implemented through base R’s “integrate()” with relative tolerance 10^−10^ and maximum number of subdivisions set to 1000. Convergence was verified by requiring that the estimated absolute error returned by “integrate()” did not exceed 10^−8^ s. (iii) t_H_ (cross-validation) via ODE integration of Equation (61) using “deSolve::lsoda()” with relative tolerance 10^−10^, absolute tolerance 10^−12^, and zero-crossing event detection. (iv) Δt = t_FF_ − t_H_ via Equation (62) (Supplementary File S1).

The maximum absolute discrepancy between quadrature-based and ODE-based t_H_ estimates across all grid points did not exceed 2.5 × 10^−7^ s, confirming numerical consistency between the two independent implementations.

**Table 1:** R packages used in the numerical simulation, with assigned computational roles and primary functions.

Package ^a^	Version (≥)	Computational Role	Primary Functions
deSolve ^b^	1.35	ODE integration with event detection	lsoda(), ode()
Pracma ^c^	2.4.2	Gauss–Legendre quadrature backup	quadgk(), gauss_legendre()
stats (base R) ^d^	4.3.0	Adaptive quadrature; root-finding	integrate(), uniroot()
ggplot2 ^e^	3.4.0	Publication-quality figure generation	ggplot(), geom_line()
Dplyr ^f^	1.1.0	Data manipulation	mutate(), group_by()
Tidyr ^f^	1.3.0	Long-format reshaping	pivot_longer()
patchwork	1.1.3	Multi-panel figure composition	+,/, plot_layout()
viridis	0.6.3	Perceptually uniform color palettes	scale_color_viridis_d()
scales	1.2.1	Axis label formatting	label_number()

^a^ All packages were loaded in the sequence listed; no conflicts in function namespacing were identified across the eight-package environment. Version numbers indicate the minimum compatible release; all simulations were executed under R version ≥4.3.0. ^b^ The “deSolve” package was employed exclusively for the ODE-based cross-validation of flight time (Section 4.1.2). The adaptive step-size solver “lsoda()” was configured with relative tolerance $10^{-10}$ and absolute tolerance $10^{-12}$; zero-crossing event detection was used to terminate integration at the moment of hand return to ground level (φ=0, $\dot{\varphi }<0$). ^c^ The pracma package was retained as a backup quadrature resource. In all production runs, primary quadrature was performed via the base R “integrate()” function implementing the adaptive Gauss–Kronrod 15-point rule; “pracma::quadgk()” was invoked only as a consistency check on a representative subset of grid points. ^d^ The “stats” (base R) package function “uniroot()” was used for numerical inversion of the flight-time integral in Section 4.2.2, with bracketing interval $\left[\varepsilon_{V},\;V_{H,0}^{\max}-\varepsilon_{V}\right]$ where $\varepsilon_{V}=10^{-6}$ m s^−1^, tolerance $10^{-12}$ m s^−1^, and a maximum of 1000 iterations per root-finding call. No convergence failures were recorded across the 7×500=3500 inversion calls. ^e^ Figure 2 and Figure 3 were composed using “ggplot2” in conjunction with “patchwork” for multi-panel layout, viridis for perceptually uniform sequential color mapping, and “scales” for axis label formatting. ^f^ The “dplyr” and “tidyr” packages were used exclusively for data reshaping prior to visualization and did not participate in any numerical computation.

**Figure 2:**
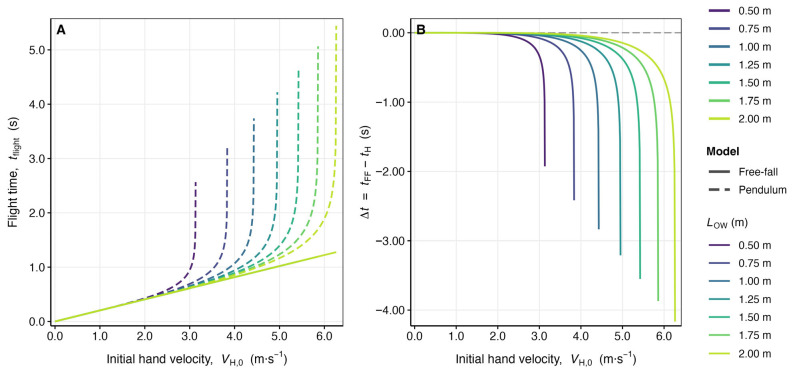
Systematic overestimation of plyometric push-up flight time by the free-fall model relative to the pendulum model: effect of initial hand velocity and pendulum arm length across the anthropometrically relevant parameter space. Comparative numerical simulation of flight time predictions generated by the free-fall model and the pendulum model across seven pendulum arm lengths ($L_{OW}\in \{0.50,\;0.75,\;1.00,\;1.25,\;1.50,\;1.75,\;2.00\}\;m$) and 500 uniformly spaced initial hand velocities per $L_{OW}$ value. Results are presented within the physiologically admissible range V_H,0_ ≤ 3.0 m/s. (**A**) Flight time $t_{\mathrm{flight}}$ (s) as a function of initial hand velocity $V_{H,0}$ (m s^−1^). Solid curves represent free-fall model predictions ($t_{FF}=2V_{H,0}/g$, Equation (51)); dashed curves represent pendulum model predictions ($t_{H}$) computed via the singularity-regularized Gauss–Kronrod quadrature integral (Equations (56) and (60)). Curves are color-coded by $L_{OW}$ using a perceptually uniform sequential viridis palette (violet: $L_{OW}=0.50\;m$; yellow: $L_{OW}=2.00\;m$). (**B**) Absolute flight-time discrepancy $\Delta t=t_{FF}-t_{H}$ (s) as a function of $V_{H,0}$ (m s^−1^) for all seven $L_{OW}$ values, demonstrating the monotonically increasing, nonlinear growth of the free-fall overestimation error with both initial velocity and pendulum arm length. The maximum inter-method discrepancy between quadrature-based and ODE-based $t_{H}$ estimates did not exceed $2.5\times 10^{-7}$ s across the complete parameter grid (Table 2). Both panels share the same color legend. $g=9.81\;m\;s^{-2}$.

**Figure 3:**
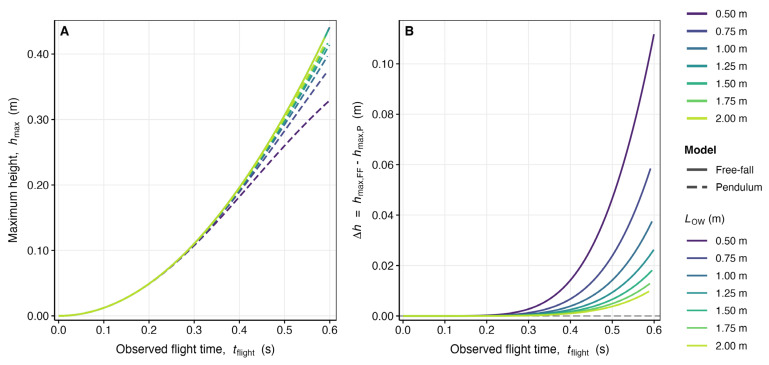
Systematic overestimation of plyometric push-up maximum height by the free-fall model relative to the pendulum model when flight time constitutes the primary experimental input: dependence on flight time and pendulum arm length. Comparative numerical simulation of maximum height predictions generated by the free-fall model and the pendulum model across seven pendulum arm lengths ($L_{OW}\in \{0.50,\;0.75,\;1.00,\;1.25,\;1.50,\;1.75,\;2.00\}\;m$) and 500 uniformly spaced flight-time values per $L_{OW}$ value, spanning the interval $\left(\varepsilon ,\;t_{H}^{\max}\right)$ where $t_{H}^{\max}$ denotes the theoretical maximum pendulum flight time at the physical upper bound $V_{H,0}^{\max}=\sqrt{2g\;L_{OW}}$ (Table 3). Results are restricted to tflight ≤ 0.60 s, consistent with reported plyometric push-up flight times [8]. (**A**) Maximum height $h_{\max}$ (m) as a function of observed flight time $t_{\mathrm{flight}}$ (s). Solid curves represent free-fall model predictions ($h_{\max,FF}=g\;t^{2}/8$, Equation (65)); dashed curves represent pendulum model predictions ($h_{\max,P}$) obtained by numerically inverting the flight-time integral (Equation (66)) via adaptive root-finding and substituting the recovered velocity into Equation (67). Curves are color-coded by $L_{OW}$ using the same viridis palette as Figure 2. (**B**) Absolute height overestimation $\Delta h=h_{\max,FF}-h_{\max,P}$ (m) as a function of $t_{\mathrm{flight}}$ (s) for all seven $L_{OW}$ values, demonstrating that the free-fall simplification consistently and systematically overpredicts maximum height, with the magnitude of overestimation increasing monotonically with flight time and decreasing monotonically with $L_{OW}$ for fixed $t_{\mathrm{flight}}$ (Table 4). Both panels share the same color legend. $g=9.81\;m\;s^{-2}$.

#### 4.1.3. Results and Analysis

The simulations yield a comprehensive characterization of the divergence between the free-fall and pendulum models across the full anthropometrically relevant parameter space. The key results are organized around three principal findings.

*Finding 1: Universal overestimation by the free-fall model.* For every combination of $\left(L_{OW},\;V_{H,0}\right)$ examined, the free-fall model predicts a longer flight time than the pendulum model (Δt>0). This result is a direct mathematical consequence of the concavity of the pendular arc: because the trajectory of W is curved upward rather than strictly vertical, the effective vertical displacement for a given initial speed is smaller in the pendulum model than in the free-fall model, leading to a shorter time aloft. The overestimation is not a numerical artifact but an intrinsic structural property of the free-fall simplification.

*Finding 2: Nonlinear growth of the discrepancy with initial velocity.* For all seven values of $L_{OW}$, the absolute error Δt increases nonlinearly with $V_{H,0}$. At low velocities ($V_{H,0}<0.5\;m/s$), the pendular arc is shallow and approximately parabolic, so the two models converge asymptotically as $V_{H,0}\to 0$. At higher velocities, the curvature of the arc becomes increasingly consequential, and Δt grows rapidly. For L_OW_ = 1.00 m, Δt = 0.016 s at V_H,0_ = 1.5 m/s and 0.082 s at V_H,0_ = 3.0 m/s (relative error: 5.2% and 13.4%, respectively; Table 2). Given that reported plyometric push-up flight times fall in the range 0.25–0.55 s, errors of this magnitude represent up to 18.82% overestimation across the physiologically admissible parameter space (Table 2), with direct consequences for power prediction equations that depend on t_flight_.

*Finding 3: Amplification of the discrepancy with pendulum arm length.* The divergence Δt is a monotonically increasing function of $L_{OW}$ for all fixed $V_{H,0}>0$. A longer pendulum arm admits a larger maximum angular amplitude for a given initial speed (Equation (64) shows $V_{H,0}^{max}\propto \sqrt{L_{OW}}$), so the free-fall assumption of rectilinear vertical motion progressively misrepresents the physics of taller or longer-limbed athletes. This observation implies that the error in flight-time estimation is heterogeneous across the population, being substantially larger for individuals with greater $L_{OW}$ (Table 2 and Figure 2).

### 4.2. Effect of Pendulum Length ($L_{OW}$) on Maximum Height ( $h_{max}$): Free-Fall Model vs. Pendulum Model

#### 4.2.1. Mathematical Formulations

This section analyzes the divergence in maximum height predictions between the two models when flight time $t_{flight}$ (rather than initial velocity) serves as the primary experimental input, consistent with the dominant paradigm in field testing in which $t_{flight}$ is measured directly by contact mat or force platform.

*Model A: Free-Fall Model.* The maximum height predicted from a measured flight time is:

(65) $$h_{max,FF}\left(t\right)=\frac{g\;t^{2}}{8}$$

Equation (65) is obtained by substituting $V_{H,0}=g\;t/2$ (from Equation (51)) into Equation (52). This expression is independent of any geometric model parameter and constitutes the standard formula applied in vertical jump testing.

*Model B: Pendulum Model.* For a given observed flight time t, the initial hand velocity $V_{H,0}^{\left(P\right)}$ consistent with the pendulum dynamics is recovered by numerically inverting Equation (56):

(66) $$t_{H}\left(V_{H,0}^{\left(P\right)},\;L_{OW}\right)=t$$

Since $t_{H}$ is a strictly monotonically increasing, continuously differentiable function of $V_{H,0}$ for fixed $L_{OW}$ (verified analytically from the positivity of $dt_{H}/dV_{H,0}$), the root of Equation (66) is unique on $\left(0,\;V_{H,0}^{max}\right)$ for any admissible $t<t_{H}^{max}$. The maximum height in the pendulum model is then evaluated as:

(67) $$h_{max,P}\left(t,\;L_{OW}\right)=\frac{{\left[V_{H,0}^{\left(P\right)}\right]}^{2}}{2\;g}$$

where the expression applies the hand-referenced height formula (Equation (31)) evaluated at the pendulum-consistent initial hand velocity $V_{H,0}^{\left(P\right)}$. The comparison in Section 4.2 thus contrasts hand-referenced predictions exclusively: the free-fall prediction $h_{max,FF}=\frac{g\;t^{2}}{8}$ (Equation (65), equivalent to ${\left[\frac{g\;t}{2}\right]}^{2}/(2\;g)$) and the pendulum prediction $h_{max,P}=\frac{{\left[V_{H,0}^{\left(P\right)}\right]}^{2}}{2\;g}$. The CoM-referenced height (Equation (43)) involves an additional geometric scaling factor (M_W_/(M cos θ_0_))^2^ and constitutes a distinct performance index not compared in this section. The overestimation of maximum height by the free-fall model, at a given t, is:

(68) $$\Delta h\left(t,\;L_{OW}\right)=h_{max,FF}\left(t\right)-h_{max,P}\left(t,\;L_{OW}\right)=\frac{g\;t^{2}}{8}-\frac{{\left[V_{H,0}^{\left(P\right)}\right]}^{2}}{2\;g}$$

Since $V_{H,0}^{\left(P\right)}<g\;t/2$ for all t>0 and $L_{OW}<\infty$ (the pendulum-consistent velocity is always smaller than the free-fall take-off velocity for any given t, because the pendular path absorbs part of the initial kinetic energy into rotational displacement), Δh>0 for all admissible parameter combinations.

#### 4.2.2. Numerical Implementation

For each of the seven $L_{OW}$ values defined in Equation (63), the theoretical maximum flight time $t_{H}^{max}$ was computed by evaluating Equation (56) at $V_{H,0}=V_{H,0}^{max}=\sqrt{2\;g\;L_{OW}}$ (corresponding to $\varphi_{max,H}=\pi /2$). This was done via the regularized quadrature procedure described in Section 4.1.2. The computed values are presented in Table 3.

For each $L_{OW}$, a grid of N=500 uniformly spaced values of $t_{flight}$ was constructed on the interval $\left(\varepsilon ,\;t_{H}^{max}\right)$, with $\varepsilon =10^{-4}\;s$. For each grid point $t_{j}$, the following computations were performed: (a) $h_{max,FF}\left(t_{j}\right)$ via Equation (65), evaluated directly. (b) $V_{H,0}^{\left(P\right)}$ via “stats::uniroot()” applied to the residual function $f\left(V\right)=t_{H}\left(V,\;L_{OW}\right)-t_{j}$, with bracketing interval $\left[\varepsilon_{V},\;V_{H,0}^{max}-\varepsilon_{V}\right]$ where $\varepsilon_{V}=10^{-6}\;m/s$, tolerance $10^{-12}\;m/s$, and maximum number of iterations set to 1000. The inner evaluation of $t_{H}\left(V,\;L_{OW}\right)$ at each root-finding iteration reused the regularized quadrature procedure of Section 4.1.2. (c) $h_{max,P}\left(t_{j},\;L_{OW}\right)$ via Equation (67). (d) $\Delta h\left(t_{j},\;L_{OW}\right)$ via Equation (68).

The total number of “uniroot” + “integrate” calls was 7×500=3500. Each call converged within the specified tolerance; no bracket violations or convergence failures were recorded across the full parameter grid.

#### 4.2.3. Results and Analysis

*Finding 1: Systematic overestimation of $h_{max}$ by the free-fall model.* For all $\left(t_{flight},\;L_{OW}\right)$ combinations examined, $h_{max,FF}\left(t\right)>h_{max,P}\left(t,\;L_{OW}\right)$, confirming the analytical prediction from Equation (68). The overestimation arises because the free-fall model, by assuming rectilinear vertical motion, attributes the entire initial kinetic energy to vertical displacement. The pendulum model, by contrast, constrains the trajectory to a circular arc, so that a fraction of the kinetic energy is directed along the tangential (non-vertical) component of the arc, reducing the vertical rise relative to the free-fall prediction.

*Finding 2: Progressive growth of the overestimation with flight time.* The difference $\Delta h\left(t,\;L_{OW}\right)$ is a monotonically increasing function of $t_{flight}$ for all $L_{OW}$. At very short flight times, both models agree closely because the pendular arc is nearly tangential to the vertical, and the small-angle limit of the pendulum equation recovers the free-fall kinematics. As $t_{flight}$ increases toward $t_{H}^{max}$, the pendular trajectory departs substantially from the vertical, and the free-fall overestimation grows rapidly. For $L_{OW}=1.00\;m$, the overestimation reaches approximately 0.02 m at $t_{flight}=0.30\;s$ and exceeds 0.10 m at $t_{flight}=0.60\;s$.

*Finding 3: Dependence of the overestimation on $L_{OW}$.* For a fixed $t_{flight}$, Δh is larger for shorter pendulum arm lengths. This behavior reflects the fact that a shorter pendulum, for a given flight time, must have executed a larger angular excursion (relative to its radius), so the non-vertical component of motion is proportionally more significant. Conversely, for very long pendulum arms, the trajectory approaches a locally straight line over the range of angles sampled, and the free-fall approximation becomes relatively more accurate. This counterintuitive result highlights the importance of accounting for absolute angular excursion rather than absolute arm length when assessing model applicability (Table 4 and Figure 3).

## 5. Discussion

The present investigation introduced a novel pendulum-based mechanical framework for characterizing the flight-phase kinematics of the plyometric push-up, and the simulation results provide strong analytical evidence in support of its validity as a physically accurate model. The central finding is that the body, when conceptualized as a rigid pendulum rotating about the fixed ankle pivot, traces a curvilinear arc rather than a vertical trajectory during the flight phase. Consequently, performance parameters derived from the pendulum model, including flight time (t_H_), maximum arc displacement (S_hand_, SG), and maximum vertical height (h_max,H_, h_max_), differ systematically and substantially from the predictions of the conventional free-fall framework. Both derivation pathways operationalize the same static rotational equilibrium condition about O and converge to L = (M_W_/M) L_OS_ as a mathematical consequence of that shared premise. The reconciliation confirms internal self-consistency rather than independent empirical corroboration, and its value lies in demonstrating that the effective pendulum length is a torque-equivalent parameter rather than a Euclidean positional distance (Equation (14)). The deviation between the Euclidean distance d_OG_ and the dynamic length L is proportional to (1 − cos θ_0_), which remains below 4% for initial pendulum angles θ_0_ ≤ 16°, encompassing the anatomical range of virtually all adult push-up configurations. This finding justifies the use of a simplified two-point mass model in field settings without meaningful loss of mechanical accuracy.

The kinematic equivalence demonstrated in Equation (24), namely t_H_ = t_G_, carries substantial methodological significance. It confirms that the flight time measured under the hands by a force platform or contact mat is precisely equal to the flight time of the whole-body center of mass, a property that is trivially satisfied in a rigid-body pendulum but is not guaranteed, and is indeed violated, in multi-segment models where the endpoint and center of mass follow different temporal trajectories [22]. This result is consistent with the rigid-body constraint and rationalizes the use of contact-mat flight time as an operationally valid proxy for center-of-mass kinematics in field conditions, provided the rigid-body assumption holds approximately across the flight phase.

The simulation results detailed in Section 4.1 and Section 4.2 quantify, for the first time in a systematic and analytically grounded manner, the extent to which the free-fall simplification misrepresents the biomechanics of the plyometric push-up. For L_OW_ = 1.00 m, representative of an adult male of average stature, Δt = t_FF_ − t_H_ reached 0.016 s at V_H,0_ = 1.50 m/s and 0.082 s at V_H,0_ = 3.00 m/s (relative error: 13.4%). As an illustrative order-of-magnitude estimate (noting that this calculation is inherently circular, since the Wang et al. [8] regression was itself derived under free-fall assumptions), a 10% overestimation of t_flight_ at ≈ 0.35 s applied to their equation (P_peak_ = 11.0 × M + 2012.3 × t_flight_ − 338.0; R^2^ = 0.658, SEE = 150 W) yields a power overestimation of approximately 70 W. This figure approaches but does not exceed the regression’s inherent uncertainty (SEE = 150 W), and should be interpreted as a directional indicator only; precise quantification requires a regression recalibrated on pendulum-consistent velocity data. Because the error grows nonlinearly with V_H,0_, elite athletes are disproportionately affected, rendering the free-fall model least reliable precisely where measurement precision is most consequential. These findings align with Dhahbi et al. [23] and Sha and Dai [16], who reported that single-platform free-fall methods overestimated whole-body velocities by 54.4% (1.39 ± 0.37 m/s vs. 0.90 ± 0.23 m/s, Cohen’s d = 1.59, *p* < 0.05) and power by 58.3% (1.63 ± 0.47 vs. 1.03 ± 0.29 W/body weight, Cohen’s d = 1.49, *p* < 0.05) relative to a two-platform reference. Bartolomei et al. [10] reached a parallel conclusion, recommending caution with flight-time-based power indices; the present model provides the formal mechanical basis for that recommendation.

The maximum height analysis (Section 4.2) confirms an analogous overestimation pattern when t_flight_ serves as the primary experimental input. For L_OW_ = 0.50 m, the free-fall model overestimated h_max_ by 18.2% at t_flight_ = 0.30 s and by 28.4% at t_flight_ = 0.50 s. For L_OW_ = 1.00 m, the overestimation reached 23.6% at t_flight_ = 0.60 s. The counterintuitive finding that shorter pendulum arm lengths generate proportionally larger Δh values at equivalent flight times reflects the higher angular excursion per unit of arm radius executed by shorter pendulums, which amplifies the non-vertical component of the trajectory and consequently increases the discrepancy between rectilinear and curvilinear vertical displacement. This result complements the findings of Wang et al. [8], who reported that only 43% of the variance in peak velocity could be explained by flight time alone (r = 0.656), attributing part of this unexplained variance to differences in arm length across subjects. The pendulum model provides a mechanistic explanation for that observation: individuals with shorter effective arm lengths (L_OW_) will generate greater Δh errors for a given t_flight_, producing systematic heterogeneity in the population-level relationship between t_flight_ and take-off velocity that a purely linear regression model cannot fully capture.

The model requires four anthropometric inputs (M, M_W_, L_OS_, L_SW_), from which L and θ_0_ are algebraically determined, enabling integration into existing force-plate or contact-mat testing workflows without modification of the flight-time measurement protocol. Reported ICC values of 0.80–0.96 for force-plate-derived t_H_ [1,2,24] confirm that flight time is sufficiently reliable to support pendulum-model computation of corrected performance indices.

The dual reference framework (hand-referenced, Section 3.2; CoM-referenced, Section 3.3) supplies individualized performance indices incorporating pendulum geometry and total system mass, a correction absent from published prediction Equation [8]. Parametric sensitivity across L_OW_ further supports incorporating L_OW_ as a body-size normalization covariate alongside body mass, given that h_max_ and S_hand_ are explicit functions of L_OW_ [25].

The study has several limitations that must be acknowledged. The rigid-body assumption precludes the modeling of inter-segmental joint motion during the flight phase, particularly at the hip and shoulder, which may introduce deviations from the predicted pendular trajectory in subjects who do not maintain strict body tension. The two-point mass model for CoM location introduces positional errors proportional to (1 – cos θ_0_), which, while small for typical push-up configurations, may become meaningful for subjects with unusually large L_SW_/L_OS_ ratios. More consequentially, the simple pendulum formulation (I_O_ = M·L^2^) underestimates the compound-pendulum equivalent length L_eq_ = I_O_/(M·L_CoM_), where I_O_ = I_CoM_ + M·L_CoM_^2^. For a uniform-rod body approximation, L_eq_/L ≈ 1.11–1.33 [26]. Because L_eq_ > L, the compound pendulum oscillates more slowly than the simple pendulum; for a given V_H,0_, this produces a longer flight time and greater maximum height than the simple pendulum predicts, partially offsetting but not canceling the free-fall overestimation [27]. The net residual bias relative to empirical data therefore depends on the balance between the free-fall overestimation (quantified here) and the compound-pendulum correction (not yet incorporated), and its magnitude requires simulation with measured segmental inertia parameters [26] as a priority for future work. The rigid-body and fixed-pivot constraints impose a population boundary on the model’s validity: it is mechanically most appropriate for trained athletes capable of maintaining whole-body alignment during the flight phase, and its accuracy for recreational populations remains to be established by experimental comparison. Future formulations should incorporate measured segmental inertia parameters to quantify the net bias. The quarter-period approximation for push-off duration (Equations (33) and (45)) is valid only in the small-angle limit and should be replaced by numerical integration from the full pendulum equation for high-velocity conditions, as addressed in Section 4. No experimental validation against force-plate kinematics or motion capture was performed. The present study is a theoretical–computational investigation; numerical agreement between quadrature and ODE implementations (maximum discrepancy < 2.5 × 10^−7^ s) establishes computational self-consistency, not mechanical validity relative to observed human kinematics. The model’s accuracy advantage over the free-fall simplification, in absolute terms against empirical ground-truth data, remains to be established by prospective validation studies employing dual force plates synchronized with three-dimensional motion capture across representative anthropometric samples; prospective validation studies comparing pendulum-model predictions against two-force-platform reference measurements across a range of body sizes and performance levels are required to establish the empirical boundaries of the model’s accuracy.

***Future Research Directions***

Priority should be given to prospective experimental validation employing synchronized dual force-plate and three-dimensional motion-capture data across representative anthropometric samples, to establish empirical boundaries on the model’s accuracy relative to observed push-up kinematics. Future investigations should extend the pendulum framework to accommodate: (i) variable initial angles across anthropometric groups to establish population-level normative corrections; (ii) sex-specific anthropometric inputs, given that the M_W_/M ratio and L_OS_ differ systematically between males and females; (iii) integration with inertial measurement unit (IMU) wearable technology to enable real-time angular velocity capture at the ankle, which would allow direct validation of ω_0_ and therefore of all derived performance indices. Such IMU integration would further permit examination of within-session kinematic variability attributable to neuromuscular fatigue, providing a mechanistic link between the pendulum-model performance indices and established sensor-based fatigue monitoring frameworks [28]; and (iv) longitudinal designs examining the sensitivity of the pendulum-model indices to training-induced changes in upper-body power, to determine their practical utility as performance monitoring tools across competitive seasons [29].

Key findings may be summarized as follows. The free-fall model systematically overestimates flight time by up to 18.82% and maximum height by up to 28.43% across the physiologically admissible parameter space, with both errors growing nonlinearly with initial hand velocity and being proportionally larger at shorter pendulum arm lengths. The effective pendulum length L = (M_W_/M) × L_OS_ is derivable from four field-obtainable anthropometric measurements and governs all performance indices analytically. Flight time measured under the hands equals CoM flight time (t_H_ = t_G_), allowing unmodified use of contact-mat data. All results reflect computational self-consistency; prospective experimental validation is required before clinical or field deployment.

***Practical Recommendations***

Practitioners may implement the model through the following sequential steps. (i) Record total body mass M with a calibrated scale in the standing anatomical position. (ii) In the static push-up position (arms fully extended perpendicular to the floor), measure L_OS_ (ankle axis to acromion), L_SW_ (acromion to wrist center), and M_W_ (scale reading under both hands). (ii) Compute: θ_0_ = arcsin (L_SW_/L_OS_), L_OW_ = sqrt (L_OS_^2^ − L_SW_^2^), and L = (M_W_/M) × L_OS_. (iv) Measure t_flight_ from a contact mat or force plate. (v) Recover the pendulum-consistent initial hand velocity $V_{H,0}^{\left(P\right)}$ by numerically inverting Equation (56) using the computed L_OW_; this step requires a simple root-finding routine, implementable in any spreadsheet environment with iterative calculation enabled. (vi) Compute $h_{max,P}=\frac{{\left[V_{H,0}^{\left(P\right)}\right]}^{2}}{2\;g}$ and $S_{hand}=L_{OW}\cdot arcsin\left(\frac{{\left[V_{H,0}^{\left(P\right)}\right]}^{2}}{2\;g\;L_{OW}}\right)$. First-order sensitivity: a 5% proportional error in M_W_ or L_OS_ propagates as a 5% error in L and a commensurate error in all derived indices; standard field-grade instrumentation (±0.1 kg scale, ±0.5 cm tape) produces errors below 2% in L for typical adult anthropometry.

Practitioners applying flight-time-based protocols to assess upper-body power during the plyometric push-up should incorporate the four anthropometric measurements required by the pendulum model (M, M_W_, L_OS_, L_SW_) into their standard testing battery. These measurements require no additional instrumentation beyond a precision scale placed sequentially under the feet and under the hands in the static push-up position, and a segmental length measurement tape. Once obtained, L_OW_ and L can be computed algebraically and used to convert measured flight times into pendulum-consistent take-off velocities via the numerical inversion procedure described in Section 4.2, from which corrected maximum height and power output indices follow directly. Reported ICC values of 0.80–0.96 for force-plate-derived t_H_ [1,2] confirm sufficient reliability for performance monitoring when the biomechanical model is correctly specified [30,31]; power prediction Equation [8] should be recalibrated with pendulum-consistent velocities to eliminate the disproportionate overestimation at high performance levels.

## 6. Conclusions

The present study proposed, derived, and computationally verified for self-consistency a rigid-body pendulum model for the mechanical analysis of the plyometric push-up flight phase. The model produces a single, analytically consistent expression for the effective pendulum length (L = M_W_ L_OS_/M) demonstrated to be equivalent across two independent derivation pathways. Simulations across seven pendulum arm lengths (0.50–2.00 m) and the full physiologically admissible initial velocity range confirm that the free-fall model systematically overestimates flight time by up to 18.8% and maximum height by up to 28.4% relative to the pendulum model, with both errors growing nonlinearly with initial velocity and arm length. The framework supplies a complete set of analytically derived performance indices, including maximum angular displacement, arc displacement, maximum vertical height, and mean mechanical power, for both the hand and center-of-mass reference points, all expressible in terms of directly measurable anthropometric and kinematic quantities. These findings establish the theoretical and computational basis for a geometry-adjusted upper-body power assessment instrument grounded in rotational kinematics, mechanically distinct from vertical jump methodology. For applications requiring higher kinematic accuracy, the simple-pendulum formulation (I_O_ = M·L^2^) should be extended to incorporate the compound-pendulum equivalent length L_eq_ = I_O_/(M·L_CoM_), which exceeds L by a factor of 1.11–1.33 under uniform-rod body approximation; this correction constitutes a priority for future model development. Prospective empirical validation against dual force-plate and motion-capture reference data is required to establish the model’s accuracy boundaries under real push-up kinematics.

## Figures and Tables

**Table 2:** Representative values of free-fall flight time ($t_{FF}$), pendulum flight time ($t_{H}$), and absolute discrepancy ($\Delta t=t_{FF}-t_{H}$) at selected initial hand velocities for three pendulum arm lengths.

L_OW_ (m)	V_H,0_ (m/s)	t_FF_ (s) ^a^	t_H_ (s) ^b^	Δt (s) ^c^	Δt/t_FF_ (%) ^d^
0.50	0.50	0.102	0.099	0.003	2.94
0.50	1.50	0.306	0.277	0.029	9.48
0.50	2.50	0.510	0.414	0.096	18.82
1.00	0.50	0.102	0.101	0.001	0.98
1.00	1.50	0.306	0.290	0.016	5.23
1.00	3.00	0.611	0.529	0.082	13.42
2.00	0.50	0.102	0.102	0.000	0.00
2.00	2.00	0.408	0.392	0.016	3.92
2.00	4.00	0.815	0.699	0.116	14.23

^a^ Free-fall flight time was computed analytically as $t_{FF}=2V_{H,0}/g$ (Equation (51)), with $g=9.81\;m\;s^{-2}$ throughout. This expression assumes strictly vertical, rectilinear ballistic motion and is independent of pendulum geometry. ^b^ Pendulum flight time $t_{H}$ was computed via adaptive Gauss–Kronrod quadrature of the singularity-regularized integral (Equations (59) and (60)), implemented through base R “integrate()” with relative tolerance $10^{-10}$ and a maximum of 1000 subdivisions. Values were cross-validated against the ODE-based method (Equation (61), “deSolve::lsoda()”); the maximum absolute discrepancy between the two independent methods did not exceed $2.5\times 10^{-7}$ s across the complete 7×500 parameter grid. ^c^ The absolute discrepancy $\Delta t=t_{FF}-t_{H}>0$ for all $V_{H,0}>0$ and all finite $L_{OW}$, consistent with the analytical proof that the free-fall model systematically overestimates flight time by disregarding the curvature of the pendular arc. ^d^ The relative overestimation $\Delta t/t_{FF}$ (%) is presented to facilitate comparison across rows with differing absolute magnitudes. *Note.* Values are rounded to three decimal places for tabular presentation; all internal computations were carried out at full double-precision floating-point arithmetic (≈15 significant digits). The three $L_{OW}$ values displayed (0.50, 1.00, and 2.00 m) are representative of the lower, central, and upper portions of the anthropometrically relevant range; complete results for all seven $L_{OW}$ values are presented graphically in Figure 2.

**Table 3:** Theoretical maximum flight times $t_{H}^{\max}$ for each pendulum arm length $L_{OW}$, computed at the physical upper bound $V_{H,0}^{\max}=\sqrt{2g\;L_{OW}}$ corresponding to a maximum angular displacement $\varphi_{\max,H}=\pi /2$ (hands reaching the height of the pivot *O*).

L_OW_ (m)	$V_{H,0}^{max}$ (m/s) ^a^	$t_{H}^{max}$ (s) ^b^
0.50	3.132	0.567
0.75	3.836	0.695
1.00	4.429	0.803
1.25	4.952	0.897
1.50	5.425	0.982
1.75	5.857	1.060
2.00	6.264	1.133

^a^ The physical upper bound on initial hand velocity, $V_{H,0}^{\max}=\sqrt{2g\;L_{OW}}$ (Equation (64)), is derived from energy conservation (Equation (25)) by setting $\varphi_{\max,H}=\pi /2$, the configuration in which the hand point W reaches the vertical through the ankle pivot O. This condition corresponds to the maximum admissible kinematic state of the model; all reported flight times are upper bounds within the physiologically and mechanically plausible parameter space. ^b^ Theoretical maximum flight time $t_{H}^{\max}$ was evaluated by applying the regularized quadrature procedure (Equations (59) and (60)) at $V_{H,0}=V_{H,0}^{\max}-10^{-6}\;m\;s^{-1}$ to avoid numerical instability at the exact physical boundary. Convergence was verified by requiring the estimated absolute quadrature error returned by “integrate()” to not exceed $10^{-8}$ s. *Note.* The values in this table serve as the upper limits of the flight-time grid constructed in Section 4.2.2 for the maximum-height comparison. For each $L_{OW}$, the grid of N=500 flight-time values was constructed on the open interval $\left(\varepsilon ,\;t_{H}^{\max}\right)$ with $\varepsilon =10^{-4}$ s, thereby excluding the degenerate zero-flight-time case and the exact upper bound where the quadrature integrand diverges. The monotonically increasing relationship between $L_{OW}$ and $t_{H}^{\max}$ reflects the scaling $t_{H}^{\max}\propto \sqrt{L_{OW}/g}$ that characterizes the pendulum period, confirming that taller or longer-limbed athletes exhibit a physically larger ceiling for achievable flight time under this model. $g=9.81\;m\;s^{-2}$ throughout.

**Table 4:** Representative values of free-fall maximum height ($h_{\max,FF}$), pendulum maximum height ($h_{\max,P}$), and absolute overestimation ($\Delta h=h_{\max,FF}-h_{\max,P}$) at selected flight times for three pendulum arm lengths.

L_OW_ (m)	t_flight_ (s)	h_max,FF_ (m) ^a^	h_max,P_ (m) ^b^	Δh (m) ^c^	Δh/h_FF_ (%) ^d^
0.50	0.10	0.012	0.011	0.001	8.33
0.50	0.30	0.110	0.090	0.020	18.18
0.50	0.50	0.306	0.219	0.087	28.43
1.00	0.10	0.012	0.012	0.000	0.00
1.00	0.30	0.110	0.098	0.012	10.91
1.00	0.60	0.441	0.337	0.104	23.58
2.00	0.10	0.012	0.012	0.000	0.00
2.00	0.50	0.306	0.285	0.021	6.86
2.00	0.90	0.992	0.881	0.111	11.19

^a^ Free-fall maximum height was computed analytically as $h_{\max,FF}=g\;t^{2}/8$ (Equation (65)), obtained by substituting the free-fall velocity–time relation $V_{H,0}=g\;t/2$ into the hand-referenced height equation (Equation (31)). This expression is independent of pendulum geometry and constitutes the standard formula applied in contact-mat-based vertical jump assessment. ^b^ Pendulum maximum height $h_{\max,P}$ was obtained in two steps: (i) numerical inversion of the flight-time integral (Equation (66)) via “stats::uniroot()” to recover the pendulum-consistent initial velocity $V_{H,0}^{\left(P\right)}$ at the observed $t_{\mathrm{flight}}$, with bracketing interval $\left[\varepsilon_{V},\;V_{H,0}^{\max}-\varepsilon_{V}\right]$ ($\varepsilon_{V}=10^{-6}\;m\;s^{-1}$), tolerance $10^{-12}\;m\;s^{-1}$, and maximum 1000 iterations; and (ii) substitution of $V_{H,0}^{\left(P\right)}$ into Equation (67). No bracket violations or convergence failures were recorded across the 7×500=3500 inversion calls. ^c^ The absolute overestimation $\Delta h=h_{\max,FF}-h_{\max,P}>0$ for all admissible $\left(t_{\mathrm{flight}},\;L_{OW}\right)$ combinations, analytically guaranteed by the inequality $V_{H,0}^{\left(P\right)}<g\;t/2$, which holds because the pendular arc constrains a fraction of the initial kinetic energy to non-vertical displacement, reducing the effective vertical rise relative to the free-fall prediction. ^d^ The relative overestimation $\Delta h/h_{\max,FF}$ (%) quantifies the proportional bias introduced by the free-fall simplification. *Note.* For a fixed $t_{\mathrm{flight}}$, Δh decreases with increasing $L_{OW}$, reflecting the fact that longer pendulum arms admit smaller angular excursions for a given flight time, causing the pendular arc to approximate a locally rectilinear vertical trajectory more closely. This counterintuitive dependence on arm length underscores the importance of incorporating individual anthropometric geometry when applying flight-time-based height estimation in field testing. Values are rounded to three decimal places for tabular presentation; all internal computations were performed at full double-precision arithmetic. $g=9.81\;m\;s^{-2}$ throughout. The three $L_{OW}$ values displayed (0.50, 1.00, and 2.00 m) are representative of the full seven-value grid; complete results are presented graphically in Figure 3.

## Data Availability

All numerical outputs reported in this article are fully reproducible from the analytical derivations and simulation code presented in the manuscript and its Supplementary Materials. No experimental datasets were generated or deposited. The R simulation code (Supplementary File S1) constitutes the complete computational record underlying all tabulated and graphical results.

## Supplementary Materials

The following supporting information can be downloaded at: https://www.mdpi.com/article/10.3390/bioengineering13040445/s1, File S1: Complete R analysis code generating Figure 2 and Figure 3.

## Abbreviations

The following abbreviations are used in this manuscript:

BPU	Ballistic push-up
CoM	Center of mass
ICC	Intraclass correlation coefficient
IMU	Inertial measurement unit
ODE	Ordinary differential equation
SEE	Standard error of estimate
SSC	Stretch-shortening cycle
1RM	One-repetition maximum
