# Supplementary material for: A Rigid-Body Pendulum Model for Plyometric Push-Up Biomechanics: Analytical Derivation and Numerical Quantification of Flight Time, Arc Displacement, Maximum Height, and Mechanical Power Output
Source: Bioengineering (Basel). 2026 Apr 11;13(4):445. doi: 10.3390/bioengineering13040445 (PMC13113941; doi:10.3390/bioengineering13040445)
Supplement: Supplementary file 1 [file bioengineering-13-00445-s001.zip › bioengineering-4227339-supplementary.pdf]

bioengineering

MDPI

Supplementary File S1

R analysis code:

```
# ==========================================================================
#
#   FIGURES 2 & 3   --   Pendulum vs. Free-Fall Model | Plyometric Push-Up
#   "A Rigid-Body Pendulum Model for Plyometric Push-Up Biomechanics"
#
#   OUTPUT    :   Figure2_FlightTime_Comparison.tiff    (600 dpi, LZW, 174 x 82 mm)
#                 Figure3_MaxHeight_Comparison.tiff     (600 dpi, LZW, 174 x 82 mm)
#                 + vector PDF counterparts
#                 All files written directly to the user Desktop.
#
#   USAGE     :   Source entire file   OR   run any selected block in RStudio.
#                 Every section is self-contained and runnable independently
#                 provided the preceding sections have been executed.
#
#   TESTED    :   R >= 4.3.0 | RStudio >= 2023.09
#
#   REVISION:   Figure 3 x-axis restricted to t_flight <= 0.60 s (physiologically
#                 admissible range per Wang et al. 2017) to resolve plotting error
#                 identified by Reviewer 2, Comment 5.
#
# ==========================================================================


# -- SECTION 0 .   Package management -----------------------------------------

pkgs <- c(
  "deSolve",      # Eq. (61): ODE solver with event/zero-crossing detection
  "ggplot2",      # Core plotting engine
  "dplyr",        # Data frame manipulation
  "tidyr",        # Wide-to-long reshaping for ggplot2
  "patchwork",    # Two-panel figure composition
  "viridis",      # Perceptually uniform, colour-blind-safe palette
  "scales"        # Axis break and label formatting
)

new_pkgs <- pkgs[!pkgs %in% rownames(installed.packages())]
if (length(new_pkgs)) install.packages(new_pkgs, dependencies = TRUE)
```

```
invisible(lapply(pkgs, library, character.only = TRUE))


# -- SECTION 1 .   Global physical constants and simulation parameters ----------

G              <- 9.81          # gravitational acceleration   (m s^-2)
N_GRID         <- 500           # evaluation points per L_OW value
EPS_V          <- 1e-4          # lower velocity guard   (m s^-1)
EPS_T          <- 1e-4          # lower time guard   (s)
REL_TOL_INT    <- 1e-10         # integrate() relative tolerance
SUBDIV         <- 1000          # integrate() maximum subdivisions
RTOL_ODE       <- 1e-10         # lsoda() relative tolerance
ATOL_ODE       <- 1e-12         # lsoda() absolute tolerance
TOL_ROOT       <- 1e-12         # uniroot() tolerance
MAXITER_ROOT <- 1000            # uniroot() maximum iterations

# Physiologically admissible upper bound for Figure 3 x-axis (Wang et al. 2017)
T_FLIGHT_MAX_PHYS <- 0.60       # s   -- applied to Figure 3 panels only

L_OW_VALS      <- c(0.50, 0.75, 1.00, 1.25, 1.50, 1.75, 2.00)     # m


# -- SECTION 2 .   Core numerical functions ------------------------------------

# -- 2.1   Singularity-regularised Gauss-Kronrod quadrature -- Eq. (56-60) -----
#
#    t_H = 2 INT_0^{phi_max} dphi / sqrt(omega0^2 - (2g/L_OW) sin(phi))
#
#    Substitution   sin(phi) = sin(phi_max) * sin^2(u)   removes the integrable
#    singularity at u = pi/2, yielding a bounded integrand for adaptive quadrature.
#
pendulum_tH_quad <- function(V_H0, L_OW) {

  omega0       <- V_H0 / L_OW
  sin_phi_max <- V_H0^2 / (2 * G * L_OW)              # Eq. (26)

  if (sin_phi_max >= 1) return(NA_real_)              # physical upper bound
  if (sin_phi_max <= .Machine$double.eps) return(0)

  integrand <- function(u) {
    sin2u   <- sin(u)^2
    sin_ph <- pmin(sin_phi_max * sin2u, 1 - 1e-15)
    cos_ph <- sqrt(1 - sin_ph^2)
```

```
    numer    <- 2 * sin_phi_max * sin(u) * cos(u)
    denom    <- cos_ph * sqrt(pmax(omega0^2 - 2 * G * sin_ph / L_OW, 0))
    ifelse(abs(denom) < 1e-15, 0, numer / denom)
  }

  out <- tryCatch(
    integrate(integrand,
              lower        = 0,
              upper        = pi / 2,
              rel.tol      = REL_TOL_INT,
              subdivisions = SUBDIV,
              stop.on.error = FALSE),
    error = function(e) list(value = NA_real_)
  )
  2 * out$value
}

# -- 2.2   ODE-based flight time -- Eq. (61) -- used for cross-validation ------
#
#    d/dt [phi, phi_dot]^T = [phi_dot, -(g/L_OW) cos(phi)]^T
#    phi(0) = 0,    phi_dot(0) = omega0
#    Integration terminated by zero-crossing event: phi(t) = 0 after take-off.
#
pendulum_tH_ode <- function(V_H0, L_OW) {

  if (V_H0^2 / (2 * G * L_OW) >= 1) return(NA_real_)

  omega0    <- V_H0 / L_OW
  t_bound <- 3.0 * (2 * V_H0 / G)
  times      <- seq(0, t_bound, length.out = 8000)

  ode_sys     <- function(t, s, p) list(c(s[2], -(G / L_OW) * cos(s[1])))
  root_fn     <- function(t, s, p) s[1]                        # zero-crossing: phi = 0

  sol <- tryCatch(
    lsoda(
      y         = c(phi = 0, dphi = omega0),
      times    = times,
      func      = ode_sys,
      parms    = NULL,
      rtol      = RTOL_ODE,
      atol      = ATOL_ODE,
      events = list(func = root_fn, root = TRUE, terminalroot = 2)
```

```
    ),
    error = function(e) NULL
  )
  if (is.null(sol)) return(NA_real_)
  max(sol[, "time"])
}

# -- 2.3   Vectorised wrappers ------------------------------------------------
vmap_quad <- function(V_vec, L)
  vapply(V_vec, pendulum_tH_quad, numeric(1), L_OW = L)

vmap_ode   <- function(V_vec, L)
  vapply(V_vec, pendulum_tH_ode,   numeric(1), L_OW = L)



# -- SECTION 3 .   Section 4.1   |   Flight-time simulation ---------------------

message("\n-- Sec. 4.1 : Flight-time grid computation ...")

df_flight <- lapply(L_OW_VALS, function(L) {

  V_max     <- sqrt(2 * G * L) - EPS_V
  V_grid    <- seq(EPS_V, V_max, length.out = N_GRID)

  t_FF      <- 2 * V_grid / G                        # Eq. (51) -- free-fall
  t_H_q     <- vmap_quad(V_grid, L)                  # Eq. (56-60) -- quadrature
  t_H_o     <- vmap_ode(V_grid, L)                   # Eq. (61)   -- ODE check

  max_disc <- max(abs(t_H_q - t_H_o), na.rm = TRUE)
  message(sprintf("     L_OW = %.2f m | V_max = %.4f | max|quad-ODE| = %.3e s",
                  L, V_max + EPS_V, max_disc))

  data.frame(
    L_OW       = L,
    V_H0       = V_grid,
    t_FF       = t_FF,
    t_H        = t_H_q,
    delta_t = t_FF - t_H_q              # Eq. (62)   Delta_t = t_FF - t_H
  )
}) |> dplyr::bind_rows() |> dplyr::filter(!is.na(t_H))

message("-- Sec. 4.1 complete.\n")
```

https://doi.org/10.3390/bioengineering13040445

```
# -- SECTION 4 .   Section 4.2   |   Maximum-height simulation -------------------

message("-- Sec. 4.2 : Maximum-height grid computation ...")

# Theoretical maximum flight times -- Eq. (64): V_H0^max = sqrt(2 * g * L_OW)
t_H_max <- vapply(L_OW_VALS, function(L)
  pendulum_tH_quad(sqrt(2 * G * L) - 1e-6, L), numeric(1))

df_height <- lapply(seq_along(L_OW_VALS), function(i) {

  L         <- L_OW_VALS[i]
  t_max     <- t_H_max[i]
  V_ceil    <- sqrt(2 * G * L) - 1e-6
  t_grid    <- seq(EPS_T, t_max - EPS_T, length.out = N_GRID)
  h_FF      <- G * t_grid^2 / 8                          # Eq. (65)

  h_P <- vapply(t_grid, function(t_obs) {

    f_lo <- pendulum_tH_quad(EPS_V, L) - t_obs
    f_hi <- pendulum_tH_quad(V_ceil, L) - t_obs
    if (is.na(f_lo) || is.na(f_hi)) return(NA_real_)
    if (f_lo >= 0 || f_hi <= 0)       return(NA_real_)

    root <- tryCatch(
      uniroot(                                          # Eq. (66) -- inversion
        f        = function(V) pendulum_tH_quad(V, L) - t_obs,
        interval = c(EPS_V, V_ceil),
        tol      = TOL_ROOT,
        maxiter  = MAXITER_ROOT
      ),
      error = function(e) NULL
    )
    if (is.null(root)) return(NA_real_)
    root$root^2 / (2 * G)                                # Eq. (67)

  }, numeric(1))

  message(sprintf("    L_OW = %.2f m | NAs: %d / %d",
                  L, sum(is.na(h_P)), N_GRID))

  data.frame(
    L_OW       = L,
```

https://doi.org/10.3390/bioengineering13040445

```
    t_flight = t_grid,
    h_FF     = h_FF,
    h_P      = h_P,
    delta_h  = h_FF - h_P                          # Eq. (68)
  )
}) |> dplyr::bind_rows() |> dplyr::filter(!is.na(h_P))

message("-- Sec. 4.2 complete.\n")


# -- SECTION 5 .  Journal theme  (Nature / J. Biomechanics / MSSE standard) ---

journal_theme <- function(base_size = 7.5) {
  theme_classic(base_size = base_size, base_family = "sans") %+replace%
    theme(
      # Axes
      axis.line        = element_line(colour = "black",  linewidth = 0.35),
      axis.ticks       = element_line(colour = "black",  linewidth = 0.30),
      axis.ticks.length = unit(2.0, "pt"),
      axis.title.x     = element_text(size = base_size,  colour = "black",
                                      margin = margin(t = 4, unit = "pt")),
      axis.title.y     = element_text(size = base_size,  colour = "black",
                                      angle = 90,
                                      margin = margin(r = 4, unit = "pt")),
      axis.text        = element_text(size = base_size - 0.5, colour = "black"),

      # Panel
      panel.background  = element_rect(fill = "white",  colour = NA),
      panel.border      = element_rect(fill = NA,       colour = "black",
                                       linewidth = 0.35),
      panel.grid.major  = element_line(colour = "grey92", linewidth = 0.25,
                                       linetype = "solid"),
      panel.grid.minor  = element_blank(),
      panel.spacing     = unit(8, "pt"),

      # Legend
      legend.position       = "right",
      legend.justification  = c(0, 0.5),
      legend.key.height     = unit(11, "pt"),
      legend.key.width      = unit(16, "pt"),
      legend.key            = element_rect(fill = NA, colour = NA),
      legend.background     = element_blank(),
      legend.box.background = element_blank(),
```

```
    legend.title         = element_text(size = base_size - 0.5,
                                       face = "bold", colour = "black"),
    legend.text          = element_text(size = base_size - 1,
                                       colour = "black"),
    legend.margin        = margin(0, 0, 0, 4, unit = "pt"),
    legend.spacing.y     = unit(1.5, "pt"),

    # Suppress all title elements (per submission requirement)
    plot.title           = element_blank(),
    plot.subtitle        = element_blank(),
    plot.caption         = element_blank(),
    plot.tag             = element_blank(),
    plot.margin          = margin(5, 5, 4, 4, unit = "pt"),
    plot.clip            = "off"
  )
}


# -- SECTION 6 .   Shared aesthetics ------------------------------------------

L_labels <- paste0(sprintf("%.2f", L_OW_VALS), " m")
n_L      <- length(L_OW_VALS)

# Viridis D -- trim endpoints for white-background legibility
pal <- viridis::viridis(n_L, option = "D", begin = 0.07, end = 0.90)

# Axis labels using plotmath -- LaTeX-equivalent in base R
lab_V    <- expression(paste(
  "Initial hand velocity,   ", italic(V)["H,0"], "   (m·s"^{-1}, ")"))
lab_t    <- expression(paste(
  "Observed flight time,   ", italic(t)["flight"], "   (s)"))
lab_tfl  <- expression(paste(
  "Flight time,   ", italic(t)["flight"], "   (s)"))
lab_Dt   <- expression(paste(
  Delta, italic(t), "   =   ",
  italic(t)["FF"], " - ", italic(t)["H"], "   (s)"))
lab_hmax <- expression(paste(
  "Maximum height,   ", italic(h)["max"], "   (m)"))
lab_Dh   <- expression(paste(
  Delta, italic(h), "   =   ",
  italic(h)["max,FF"], " - ", italic(h)["max,P"], "   (m)"))

# Panel-tag annotator -- bold letter inset, upper-left
```

https://doi.org/10.3390/bioengineering13040445

```
tag <- function(label) {
  annotate("text",
           x = -Inf, y = Inf,
           label    = label,
           hjust    = -0.55, vjust = 1.65,
           fontface = "bold",
           size     = 2.9,
           colour   = "black",
           family   = "sans")
}

# Reference line at Delta = 0
zero_line <- geom_hline(
  yintercept = 0,
  linetype   = "longdash",
  colour     = "grey50",
  linewidth  = 0.30
)

# Linetype lookup for the two models
lty_map <- c("Free-fall" = "solid", "Pendulum" = "42")



# -- SECTION 7 .   Figure 2 ----------------------------------------------------
#    Panel A :   t_flight   vs.   V_H,0              (free-fall solid, pendulum dashed)
#    Panel B :   Delta_t = t_FF - t_H   vs.   V_H,0   (per L_OW, colour-coded)
#    NOTE: Figure 2 retains the full velocity range (no physiological cap applied)
#              because the x-axis is initial velocity, not flight time.

# -- 7.1   Reshape for Panel A ------------------------------------------------
df_f_long <- df_flight |>
  dplyr::mutate(
    L_label = factor(paste0(sprintf("%.2f", L_OW), " m"), levels = L_labels)
  ) |>
  tidyr::pivot_longer(
    cols      = c(t_FF, t_H),
    names_to  = "Model",
    values_to = "t_flight"
  ) |>
  dplyr::mutate(
    Model = factor(
      dplyr::recode(Model, "t_FF" = "Free-fall", "t_H" = "Pendulum"),
      levels = c("Free-fall", "Pendulum")
```

https://doi.org/10.3390/bioengineering13040445

```
    )
  )

df_f_disc <- df_flight |>
  dplyr::mutate(
    L_label = factor(paste0(sprintf("%.2f", L_OW), " m"), levels = L_labels)
  )

# -- 7.2   Panel A -----------------------------------------------------------
p2A <- ggplot(df_f_long,
              aes(x = V_H0, y = t_flight,
                  colour    = L_label,
                  linetype = Model)) +
  geom_line(linewidth = 0.50, alpha = 0.95) +
  scale_colour_manual(
    name     = expression(italic(L)["OW"]*" (m)"),
    values = pal,
    guide   = guide_legend(
      title.position = "top",
      ncol                  = 1,
      keywidth            = unit(16, "pt"),
      override.aes     = list(linetype = "solid", linewidth = 0.75)
    )
  ) +
  scale_linetype_manual(
    name     = "Model",
    values = lty_map,
    guide   = guide_legend(
      title.position = "top",
      ncol                  = 1,
      keywidth            = unit(16, "pt"),
      override.aes     = list(colour = "grey25", linewidth = 0.75)
    )
  ) +
  scale_x_continuous(
    name     = lab_V,
    breaks = scales::pretty_breaks(n = 5),
    labels = scales::label_number(accuracy = 0.5),
    expand = expansion(mult = c(0.015, 0.025))
  ) +
  scale_y_continuous(
    name     = lab_tfl,
    breaks = scales::pretty_breaks(n = 5),
```

```
      labels = scales::label_number(accuracy = 0.1),
      expand = expansion(mult = c(0.015, 0.025))
   ) +
   tag("A") +
   journal_theme()

# -- 7.3    Panel B ------------------------------------------------------------
p2B <- ggplot(df_f_disc,
                     aes(x = V_H0, y = delta_t, colour = L_label)) +
   zero_line +
   geom_line(linewidth = 0.50, alpha = 0.95) +
   scale_colour_manual(
      name      = expression(italic(L)["OW"]*" (m)"),
      values = pal,
      guide    = guide_legend(
         title.position = "top",
         ncol                  = 1,
         keywidth            = unit(16, "pt")
      )
   ) +
   scale_x_continuous(
      name      = lab_V,
      breaks = scales::pretty_breaks(n = 5),
      labels = scales::label_number(accuracy = 0.5),
      expand = expansion(mult = c(0.015, 0.025))
   ) +
   scale_y_continuous(
      name      = lab_Dt,
      breaks = scales::pretty_breaks(n = 5),
      labels = scales::label_number(accuracy = 0.01),
      expand = expansion(mult = c(0.015, 0.050))
   ) +
   tag("B") +
   journal_theme() +
   theme(legend.position = "none")

# -- 7.4    Compose ------------------------------------------------------------
fig2 <- (p2A | p2B) +
   plot_layout(guides = "collect", widths = c(1.08, 1.00)) &
   theme(legend.position = "right")


# -- SECTION 8 .    Figure 3 ----------------------------------------------------
```

https://doi.org/10.3390/bioengineering13040445

```
#   Panel A :  h_max  vs.  t_flight        (free-fall solid, pendulum dashed)
#   Panel B :  Delta_h = h_FF - h_P  vs.  t_flight  (per L_OW, colour-coded)
#
#   FIX (Reviewer 2, Comment 5): both panels are restricted to
#   t_flight <= T_FLIGHT_MAX_PHYS (0.60 s) by filtering df_height before
#   reshaping. This resolves the x-axis extension to 6.0 s in the original
#   figure, which exceeded the physiologically admissible range stated in
#   the caption. The underlying df_height data (used for Table 4) is unchanged.

# -- 8.1  Filter to physiologically admissible range -------------------------
df_height_phys <- df_height |>
  dplyr::filter(t_flight <= T_FLIGHT_MAX_PHYS)

# -- 8.2  Reshape for Panel A ------------------------------------------------
df_h_long <- df_height_phys |>
  dplyr::mutate(
    L_label = factor(paste0(sprintf("%.2f", L_OW), " m"), levels = L_labels)
  ) |>
  tidyr::pivot_longer(
    cols      = c(h_FF, h_P),
    names_to  = "Model",
    values_to = "h_max"
  ) |>
  dplyr::mutate(
    Model = factor(
      dplyr::recode(Model, "h_FF" = "Free-fall", "h_P" = "Pendulum"),
      levels = c("Free-fall", "Pendulum")
    )
  )

df_h_disc <- df_height_phys |>
  dplyr::mutate(
    L_label = factor(paste0(sprintf("%.2f", L_OW), " m"), levels = L_labels)
  )

# -- 8.3  Panel A ------------------------------------------------------------
p3A <- ggplot(df_h_long,
              aes(x = t_flight, y = h_max,
                  colour   = L_label,
                  linetype = Model)) +
  geom_line(linewidth = 0.50, alpha = 0.95) +
  scale_colour_manual(
    name    = expression(italic(L)["OW"]*" (m)"),
```

```
    values = pal,
    guide    = guide_legend(
      title.position = "top",
      ncol              = 1,
      keywidth          = unit(16, "pt"),
      override.aes      = list(linetype = "solid", linewidth = 0.75)
    )
  ) +
  scale_linetype_manual(
    name      = "Model",
    values = lty_map,
    guide    = guide_legend(
      title.position = "top",
      ncol              = 1,
      keywidth          = unit(16, "pt"),
      override.aes      = list(colour = "grey25", linewidth = 0.75)
    )
  ) +
  scale_x_continuous(
    name      = lab_t,
    limits = c(0, T_FLIGHT_MAX_PHYS),
    breaks = scales::pretty_breaks(n = 5),
    labels = scales::label_number(accuracy = 0.1),
    expand = expansion(mult = c(0.015, 0.025))
  ) +
  scale_y_continuous(
    name      = lab_hmax,
    breaks = scales::pretty_breaks(n = 5),
    labels = scales::label_number(accuracy = 0.01),
    expand = expansion(mult = c(0.015, 0.025))
  ) +
  tag("A") +
  journal_theme()

# -- 8.4    Panel B ------------------------------------------------------------
p3B <- ggplot(df_h_disc,
                    aes(x = t_flight, y = delta_h, colour = L_label)) +
  zero_line +
  geom_line(linewidth = 0.50, alpha = 0.95) +
  scale_colour_manual(
    name      = expression(italic(L)["OW"]*" (m)"),
    values = pal,
    guide    = guide_legend(
```

```
            title.position = "top",
            ncol                   = 1,
            keywidth            = unit(16, "pt")
        )
    ) +
    scale_x_continuous(
        name      = lab_t,
        limits = c(0, T_FLIGHT_MAX_PHYS),
        breaks = scales::pretty_breaks(n = 5),
        labels = scales::label_number(accuracy = 0.1),
        expand = expansion(mult = c(0.015, 0.025))
    ) +
    scale_y_continuous(
        name      = lab_Dh,
        breaks = scales::pretty_breaks(n = 5),
        labels = scales::label_number(accuracy = 0.01),
        expand = expansion(mult = c(0.015, 0.050))
    ) +
    tag("B") +
    journal_theme() +
    theme(legend.position = "none")

# -- 8.5    Compose ------------------------------------------------------------
fig3 <- (p3A | p3B) +
    plot_layout(guides = "collect", widths = c(1.08, 1.00)) &
    theme(legend.position = "right")



# -- SECTION 9 .    Export to Desktop -------------------------------------------
#
#     Specification:
#         TIFF    : 600 dpi, LZW compression    -- primary submission format
#         PDF      : Cairo vector                         -- supplementary / press-ready format
#         Size    : 174 x 82 mm                         -- double-column width per most Q1 journals
#                           (Elsevier / Springer / Nature guidelines)

desktop_path <- if (.Platform$OS.type == "windows") {
    file.path(Sys.getenv("USERPROFILE"), "Desktop")
} else {
    file.path(path.expand("~"), "Desktop")
}
if (!dir.exists(desktop_path)) desktop_path <- path.expand("~")
```

https://doi.org/10.3390/bioengineering13040445

```
FIG_W_MM   <- 174
FIG_H_MM   <- 82
DPI        <- 600
FIG_W_IN   <- FIG_W_MM / 25.4
FIG_H_IN   <- FIG_H_MM / 25.4

# -- TIFF exports -------------------------------------------------------------
ggsave(
  filename    = file.path(desktop_path, "Figure2_FlightTime_Comparison.tiff"),
  plot        = fig2,
  device      = "tiff",
  width       = FIG_W_MM, height = FIG_H_MM, units = "mm",
  dpi         = DPI,
  compression = "lzw",
  bg          = "white"
)
message(sprintf("\u2713  Figure 2 TIFF  ->  %s",
                file.path(desktop_path, "Figure2_FlightTime_Comparison.tiff")))

ggsave(
  filename    = file.path(desktop_path, "Figure3_MaxHeight_Comparison.tiff"),
  plot        = fig3,
  device      = "tiff",
  width       = FIG_W_MM, height = FIG_H_MM, units = "mm",
  dpi         = DPI,
  compression = "lzw",
  bg          = "white"
)
message(sprintf("\u2713  Figure 3 TIFF  ->  %s",
                file.path(desktop_path, "Figure3_MaxHeight_Comparison.tiff")))

# -- PDF (Cairo vector) exports -----------------------------------------------
ggsave(
  filename = file.path(desktop_path, "Figure2_FlightTime_Comparison.pdf"),
  plot     = fig2,
  device   = cairo_pdf,
  width    = FIG_W_IN, height = FIG_H_IN, units = "in",
  bg       = "white"
)
message(sprintf("\u2713  Figure 2 PDF   ->  %s",
                file.path(desktop_path, "Figure2_FlightTime_Comparison.pdf")))

ggsave(
```

```
    filename = file.path(desktop_path, "Figure3_MaxHeight_Comparison.pdf"),
    plot     = fig3,
    device   = cairo_pdf,
    width    = FIG_W_IN, height = FIG_H_IN, units = "in",
    bg       = "white"
)
message(sprintf("\u2713    Figure 3 PDF     ->    %s",
                file.path(desktop_path, "Figure3_MaxHeight_Comparison.pdf")))

message("\n=== ALL EXPORTS COMPLETE ===\n")

# -- Optional: render interactively in the RStudio Plots pane -----------------
# print(fig2)
# print(fig3)
```

https://doi.org/10.3390/bioengineering13040445
